# Decadal-scale variation in diet forecasts persistently poor breeding under ocean warming in a tropical seabird

**DOI:** 10.1371/journal.pone.0182545

**Published:** 2017-08-23

**Authors:** Emily M. Tompkins, Howard M. Townsend, David J. Anderson

**Affiliations:** 1 Department of Biology, Wake Forest University, Winston-Salem, North Carolina, United States of America; 2 NOAA/NMFS/HC/Chesapeake Bay Office, Cooperative Oxford Lab, Oxford, Maryland, United States of America; Technical University of Denmark, DENMARK

## Abstract

Climate change effects on population dynamics of natural populations are well documented at higher latitudes, where relatively rapid warming illuminates cause-effect relationships, but not in the tropics and especially the marine tropics, where warming has been slow. Here we forecast the indirect effect of ocean warming on a top predator, Nazca boobies in the equatorial Galápagos Islands, where rising water temperature is expected to exceed the upper thermal tolerance of a key prey item in the future, severely reducing its availability within the boobies’ foraging envelope. From 1983 to 1997 boobies ate mostly sardines, a densely aggregated, highly nutritious food. From 1997 until the present, flying fish, a lower quality food, replaced sardines. Breeding success under the poor diet fell dramatically, causing the population growth rate to fall below 1, indicating a shrinking population. Population growth may not recover: rapid future warming is predicted around Galápagos, usually exceeding the upper lethal temperature and maximum spawning temperature of sardines within 100 years, displacing them permanently from the boobies’ island-constrained foraging range. This provides rare evidence of the effect of ocean warming on a tropical marine vertebrate.

## Introduction

Long-term studies of animal populations have provided crucial evidence regarding effects of climate change on population size, abundance, and future viability [1–4]. However, longitudinal studies at the population level often cannot effectively evaluate the proximate factors affecting population size under climate change, because disentangling the separate effects of demographic vital rates (breeding, survival, emigration, immigration) requires long-term individual-based data [5–7]. Connecting vital rates with climate to reach a mechanistic understanding of a natural population’s vulnerability requires experimental approaches [8,9], or, since experimental manipulations are often impractical, data on survival, breeding, and movement under contemporary climate change and variability [6,10]. Important progress has been made using this second method in long-term studies of identifiable individuals at locations where climate change is already rapid, permitting evaluation of cause-effect relationships in real time (e.g., [11–14]). This enables robust projections of species’ responses to climate change for these areas, but leaves significant gaps in our mechanistic understanding of population viability under climate warming for tropical animals because warming has been slow in much of the tropics [15–17]. Tropical systems are greatly underrepresented in climate change studies [18–21], as are marine systems [22–23]. At the intersection of these two knowledge gaps, increasing our understanding of species’ responses to climate change in the tropical oceans is a high priority [18,24].

The very high velocity and impact of climate change expected in much of the tropics [16,25,26], particularly the tropical oceans, makes this geographic bias especially noteworthy. Top predators may face stiff challenges in the tropics because their generation times are typically long, so they have little capacity for adaptive evolution on the time scale required by rapid climate change [27]. Instead, many long-lived predators must cope with climate change chiefly through plastic phenotypic adjustments in real time, within a few generations. Aspects of their phenotypes, such as life history characteristics and diet composition, must be flexible enough that new combinations of vital rates and ecological interactions avoid negative population growth [12].

Here we exploit contemporary decadal-scale variation in diet and breeding of a tropical pelagic predator (the Nazca booby, *Sula granti*) in the Galápagos Islands to forecast the effect of future exclusion, by ocean warming, of a key prey species on this seabird’s population growth rate. Climate change will manifest in a variety of ways relevant to seabird populations. These include abiotic effects of precipitation, ocean acidification, sea level rise, and indirect biotic effects mediated by trophic relationships and other interspecific interactions. We consider regional increase in sea surface temperature and ensuing trophic effects on Nazca boobies under ocean warming, recognizing that climate change in this region will probably involve many additional effects on tropical seabirds, including altered frequency of extremes in climate such as *El Niño* events. There is growing recognition that disrupted biotic interactions [28] are a main determinant of population responses of long-lived predators to climate change [29–31]. We focus on indirect thermal effects reflected by sea surface temperature (SST) because recent refinements of ocean-atmosphere coupled global climate models indicate that the ocean water in the eastern tropical Pacific (ETP) around Galápagos will become 4.5°C warmer over the next 100 years. This rate exceeds that of almost all other regions of the world’s oceans [32,33]. Such a dramatic change in mean SST will probably make a critical prey species, Pacific sardines (*Sardinops sagax*; [34]), functionally absent from this top predator’s central-place foraging range around Galápagos.

**Fig 1:**
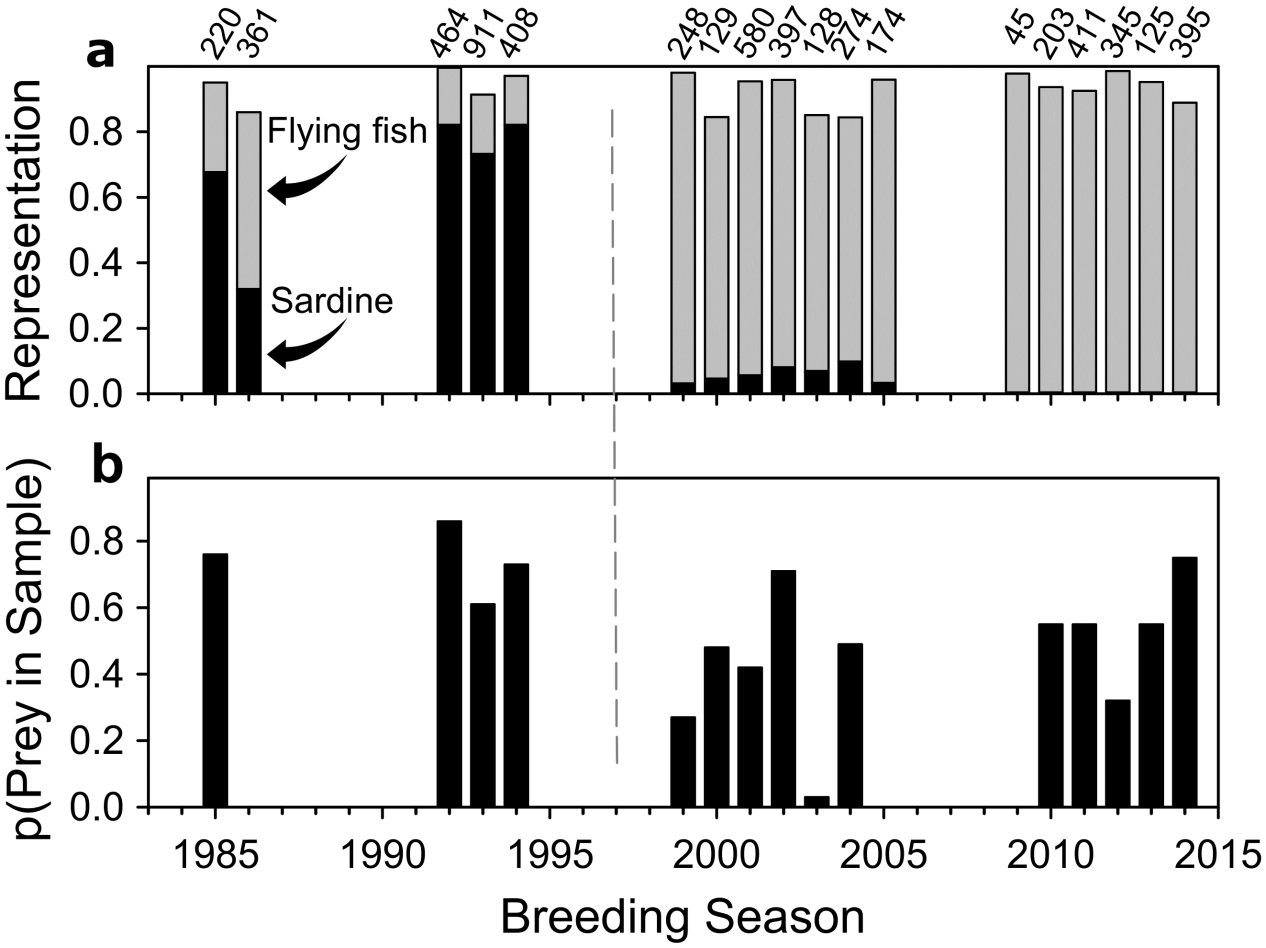
Temporal change in diet of Nazca boobies. (a) Numerical representation of the two major prey types in systematic and opportunistic regurgitation samples. Missing years due to permit restrictions (mid-2000s) or to lack of data (1980s and mid-1990s; sardines (black fill) dominated casual observations during these gaps until the 1997–98 *El Niño* event [dashed line] but data not recorded). # prey items at top of bar. Breeding spans two calendar years (October-May), “breeding seasons” are labelled by the first year in each two-year spread. Black and gray bars do not sum to 1.0 because sardines and flying fish are not the only prey types in the diet. (b) Probability of a bird producing at least one prey item in 1,345 systematic regurgitation samples collected in January-April (probability is lower after 1997–98; Mann-Whitney U Test, N_1_ = 4, N_2_ = 11, Z = 2.48, *P* = 0.01; see S1 Methods).

**Fig 2:**
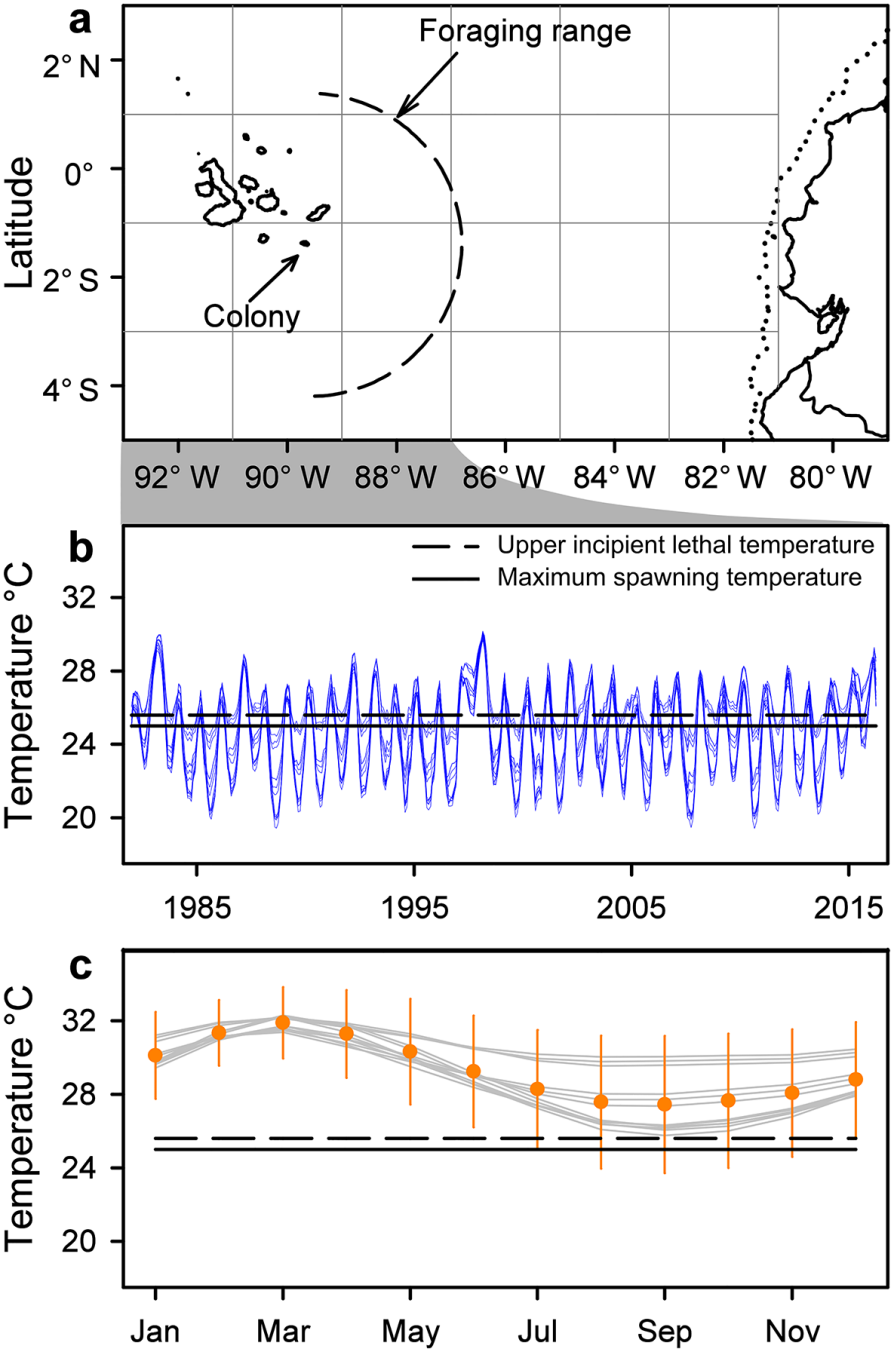
Recent SST + 4.5°C, sardine spawning temperature, and Nazca booby foraging range. (a) Foraging envelope [48] (semicircle) of breeding Nazca boobies from Isla Española, Galápagos. Most present-day trips are within the area of the semicircle. The potential foraging range, including west of the colony, includes parts of each of the western-most 12 blocks on the map. Dotted line to the east shows the continental shelf break (1000 m isobath), roughly indicating the distribution of continental Pacific sardines, a probable source population for Galápagos. (b) Recent temporal variation (blue lines) in SST in each of 12 2° x 2° blocks within the present-day foraging range of Nazca boobies. Solid horizontal line shows upper limit of spawning SST range of Pacific sardines (25°C; [45–47]). Dashed horizontal line shows upper incipient lethal limit for warm-acclimated temperate-origin Pacific sardines (25.6°C; [44]). (c) Recent SST from (a) with 4.5°C warming expected within 100 years. Orange pts (± 2 S.D.) show expected temperature averaged across all 12 blocks and across all years (1982–2016) by month. Gray lines show temperatures averaged across all years, by month, for each individual block. Monthly SST values for each 2° x 2° block were downloaded on 12 May 2016 from http://iridl.ldeo.columbia.edu/SOURCES/.NOAA/.NCDC/.ERSST/.version3b/.sst/.

Rising ocean temperatures are expected to move distributions of many marine fish pole-ward [35]. This is especially true of small-bodied species like Pacific sardines with short generation times [35,36], and at the margin of a distribution [36] such as the peripheral population of this species in the equatorial Galápagos [37]. Pacific sardines are lipid-rich schooling forage fish [38–40], prominent in the diets of Nazca boobies (Fig 1), Galápagos blue-footed boobies (*S*. *nebouxii*; [34]), and Galápagos sea lions (*Zalophus wollebaeki*; [41]) until the 1997–98 *El Niño* event. The provenance of these prey is unknown. Pacific sardines are capable of migrating or dispersing significant distances [42]. Any such movements from the large possible source populations in the thermally temperate Peruvian Upwelling should stop within 100 years because the SST expected around Galápagos will usually exceed the “upper incipient lethal temperature” (the maximum temperature that can be tolerated indefinitely [43]) of adult, warm-adapted, temperate-origin Pacific sardines [44] (Fig 2, S1 Fig). Similarly, if the population were local, self-sustaining, and tropical-origin and could tolerate high SST, it will live in waters at temperatures typically above the maximum spawning temperature of the species [45–47] (Fig 2, S1 Fig). Regardless of the provenance of these prey, thermal constraints are expected to severely reduce their availability or exclude them from the boobies’ diet within 100 years.

Food controls the breeding success of tropical seabirds (other environmental factors like predators and weather usually have negligible direct effects [49,50]), so contemporary variation in sardine availability affords a window into the future for Nazca boobies. After 1997 sardines became rare in the diets of sea lions and both booby species ([51–53]; Fig 1). Lacking these valuable prey, the Galápagos population of blue-footed boobies (a sardine specialist [34]) declined by approximately 2/3 because breeding performance is linked to sardine availability [52]. This dramatic demographic effect motivated our use of time series data on diet and longitudinal data on breeding of individually marked, known-age Nazca boobies to test the idea that a more flexible predator [34] can maintain breeding success and population viability when sardines are not available. This analysis parallels the demographic approach commonly used at higher latitudes (e.g., [13,54]), taking advantage of a qualitative change in a key ecological relationship that anticipates the relationship expected under future ocean warming.

## Materials and methods

### Data collection

We studied diet, breeding, and survival of Nazca boobies as part of a long-term study at Punta Cevallos, Isla Española, Galápagos Islands (1°20’ S, 89°40’ W; see [55,56] for details of the site) from the 1983–84 breeding season until the 2012–13 season. Nazca boobies in our population breed seasonally from October to June, with most egg laying completed by January, raising no more than one offspring to independence per annual breeding season. We refer to breeding seasons by the calendar year in which the season began. Marking of adults and young of the year with uniquely numbered metal leg bands began in 1984. Young of the year were banded in 1984–1987 and 1992–2012 and analyses restricted to known-age birds use these individuals. In most years some birds were banded as adults. These adults are considered to be at least 4 years old when banded because 4 years is the median age at recruitment [57].

We monitored annual breeding parameters of banded adults in all years of the study except 1987–1991. Newly independent young leave Galapagos for 2–7 years before recruiting to the breeding colony [57]. An individual was considered an “adult”, and therefore a potential breeder, from its year of recruitment until its “death” (see below). “Annual Breeding Success” is our primary dependent variable for breeding and is defined as the production of an independent offspring (yes/no) given that the adult was alive. Annual Breeding Success was scored as “1” for a given parent in a given year if that parent had an offspring late in the breeding season and we did not find its offspring dead at the nest site or (noting its band number) elsewhere. Daily nest monitoring for all banded birds in some years allowed finer grained assessment at sequential reproductive stages, all of which were similarly dichotomous: laying of at least one egg given that the bird was alive (“initiate a clutch | alive”), hatching of one or more eggs given that a clutch was initiated (“hatch an egg | clutch was initiated”), and production of an independent offspring given that at least one egg hatched (“produce independent offspring | hatch”). Re-nesting occurs only if the first clutch fails, and we did not distinguish between hatchlings produced or offspring reaching independence from an original clutch *versus* from a re-nest clutch. Annual Breeding Success was monitored for all banded birds from 1992–2012 except 2007, when only a subset of banded individuals was tracked. Complete data on all reproductive stages were available for the period 1992–2004. Seasons 2005–2009 lack comprehensive monitoring of clutch initiation and hatching success, and 2010–2012 lack comprehensive information on hatching success. All analyses excluded rare adoption cases (adoptions affect only 1.6% of clutches) and one anomalous record when a pair raised two offspring.

Annual survival and recapture data came from nighttime band resight surveys (“BRS”) conducted early in each season in 1984–2014 except 1988 [58]. We considered a bird dead (and so no longer a breeder) in the first year of a series of two or more years in which it did not appear in the BRS. Resight probabilities in our population are essentially 1 for breeders. Recapture rates are lower and more variable for nonbreeders [58]. However, only 1% of birds that were absent in two consecutive years ever reappear in our BRS dataset. “Death” is unlikely to be confounded with emigration because Nazca boobies are highly philopatric and show high colony fidelity after recruiting to their natal colony [55].

### Statistical analyses

The effect of diet on the breeding success of male and female Nazca boobies was first examined in detail across the successive stages of a breeding attempt (laying eggs, hatching eggs, raising offspring). Young, middle-aged, and old breeders may respond differentially to environmental challenges (e.g., [59,60]). Such age by environment interactions contribute to our understanding of life-history variation in the wild and may critically affect population dynamics [61]. Therefore, these analyses focused on interactions between diet and age, as well as on the main effects of diet on breeding performance in Nazca boobies. Environmental variation impacts population viability through effects on vital rates. Links between environmental variation and survival, reproductive success, and juvenile recruitment may not agree in strength or sign (e.g., [11,62]) so that consideration of multiple vital rates is prerequisite to understanding the total effect of an environmental parameter of interest. Taking this into consideration, we parameterized matrix population models with data on Nazca booby reproduction, juvenile survival, and adult survival to evaluate the effect of high- and low-quality diet on population growth rate.

#### Modeling annual breeding parameters

We modeled variation in annual breeding parameters for males and females separately using generalized linear mixed effect models (GLMMs) with binomial error structure and a logit link function. Crossed random effects of individual identity and year were included to account for the non-independence of repeated measures on the same individual and the non-independence of data points subject to the same environmental conditions within a year. To accommodate expected age effects on performance, and to evaluate any interaction between diet and age, we modeled breeding performance in young to middle-age birds (ages ≤ 11) and then, separately, in presumed-old birds. Our banding program had not yet produced known-age members of the oldest age classes by the end of the Sardine Phase (1984–1996) to compare with the complete known age structure from the Flying Fish Phase (1997–2014). Accordingly, to compare late-life performance under the contrasting diets we relied on banded individuals of unknown age (leg-banded as adults) and used years before death (YBD) as a proxy for chronological age [63]. To improve the proxy’s representation of the performance of old individuals, we included only birds whose age at death was known to have been ≥13 yrs and modeled changes in performance during the final five years before death. This, coupled with very high annual survival until beyond the age of successful breeding (S2 and S3 Figs; of the 58% of recruits alive at age 13, 47% are still alive at age 20), validates the use of this proxy.

Global models, run separately for young and middle-aged and for old birds, for each sex/trait combination included Fish Phase modeled as a binary factor dividing years pre- and post-1997. Models included up to three covariates to capture further environmental effects. A binary factor identified the extreme 1997–98 *El Niño* Southern Oscillation warm event in models for young to middle-aged birds; no presumed-old bird raised a chick during the *El Niño* and so this breeding season was excluded from models for old birds. Two linear continuous covariates modeled fine-scale inter-annual variation in sea surface temperature anomaly (SSTA). Monthly SSTA was averaged across December-February (SSTA_DJF_) to represent the intensity of annual events [64]. Monthly SSTA was averaged across April-June (SSTA_AMJ_) to represent environmental conditions during chick rearing. SSTA_AMJ_ was excluded from global models describing variation in probability of nest initiation (which is completed long before April). SSTA_DJF_ and SSTA_AMJ_ over the entire period of study (1984–2014) are not correlated (*r* = 0.32, df = 28, *P* = 0.08) and showed no linear temporal trends (SSTA_DJF_: β = 0.004, SE = 0.02, *P* = 0.85; SSTA_AMJ_: β = 0.01, SE = 0.02, *P* = 0.83; [65]). Monthly SSTA values for *El Niño* Region 3 (latitude 5° S to 5° N and longitude 150° W to 90° W) were obtained from the LDEO/IRI Data Library at: http://iridl.ldeo.columbia.edu/SOURCES/.NOAA/.NCEP/.EMC/.CMB/.GLOBAL/.Reyn_SmithOIv2/.monthly/ (accessed on 07/20/2015).

Global models for young and middle-age birds included parent age fit as linear and quadratic terms and interacting with Fish Phase. Global models for presumed-old birds included YBD fit as a linear term and interacting with Fish Phase. Although we modelled the sexes separately, highly correlated ages of mates may enhance or obscure age-related performance changes in one sex by correlated age-related changes in the other sex. Mate rotation in this species [66,67] decouples pair identities and ages, allowing independent estimation of male and female aging trends. For all known-age pairs in our long-term dataset, the correlation between male and female ages is low but reaches statistical significance (*r* = 0.255, df = 3,932, *P* < 0.001), as does the correlation between male and female YBD (*r* = 0.092, df = 2,466, *P* < 0.001). Adding partner age/YBD to models for each sex gave no indication that the low correlation between male and female age/YBD affected estimation of sex-specific effects (results not shown). Population-level age effects may confound individual aging trends with selective appearance or disappearance [68,69]. We did not attempt to model selective appearance. The use of YBD to model senescence circumvents the issue of selective disappearance in old birds [63].

Taking the global model for each sex/trait combination, we constructed a model set containing all possible additive combinations of the explanatory terms, with the restriction that any model including a higher order term (e.g., a quadratic or an interaction term) must include the relevant lower order terms. We used Akaike’s Information Criterion corrected for small sample size (AICc) for model selection and ranking [70]. Competing models within 2 AICc units of the “top model” (that with the lowest AICc value) are all considered strongly supported, while models greater than 9–11 ΔAICc units from the top model have relatively low support [71]. We present model rankings for the subset of models within ΔAICc of 7 of the top model, and we highlight cases where multiple models are strongly supported (ΔAICc ≤ 2), such that there is no single top model (S1–S4 Tables). We disregard models in the top set that are more complex nested versions of models with lower AICc values (*sensu* ref. [72]). We make predictions from, and report the coefficients of, the top model in the set. We infer a parameter’s importance from its representation among those top models and the span of the 95% CI surrounding the parameter, calculated using a parametric bootstrap (1,000 samples). All models were run in R (v. 3.1.3; [73]) using package lme4 [74]. Model selection used package MuMIn [75]. Sample sizes for each sex/trait combination are listed in S7 and S8 Tables.

#### Population growth rate

We built single-sex, age-structured, population projection matrices [76] from female fertility and survival parameters based on birth-pulse dynamics and an annual pre-breeding survival census. Initial modeling of survival and reproduction indicated substantial inter-annual variability in demographic rates within each Fish Phase (S2 and S4 Figs), so we constructed an individual matrix for each breeding season 1992–1996 (in the Sardine Phase), the 1997 *El Niño* event, and 1998–2012 (in the Flying Fish Phase), referred to as “annual projection matrices”. The annual projection matrices were set up with 22 age classes to accommodate the oldest successful breeders in our long-term dataset; the first 21 age classes span ages 1–22, and the final age class included all ages ≥ 22. The fertility coefficient, F_*i*_, for each age class *i* = 3–21 was defined as the mean number of daughters surviving until the next BRS per female in age class *i*. F_*i*_ for age classes 1, 2, and 22 were set to zero, based on field data. Fertility coefficients incorporate the proportion of living females who breed, the mean number of offspring produced by those females, and mean offspring survival during the first year. This last value required an assumption about the timing of death in pre-breeding Nazca boobies, who may not attend the colony, and would be undetectable, for the first 3–6 yrs following independence [57]. We made the simplifying assumption that mortality between independence and breeding occurs soon after independence, when young of the year are inexperienced. Thus, we incorporate survival of the juvenile period into fertility coefficients and set the survival coefficient, P_*i*_, for age classes *i* = 1–3 to one. Note that fertility for age classes < 4 is zero or near zero, so that underestimation of first year survival and corresponding overestimation of survival until age 4 do not alter population dynamics.

We generate F_*i*_ for each annual projection matrix in two stages. First, we evaluated the combined probabilities that a living female will breed, that she will produce an offspring, and that the offspring will survive until independence, in a single model predicting Annual Breeding Success by female age and year. Female age and year were fit additively as multi-level factors in a GLMM with a random effect of female identity and binomial errors (logit link, N = 1,543 records from 2,543 known-age females during breeding seasons 1992–2006, 2008–2012). The F_*i*_ count only female offspring, so final age- and year-specific Annual Breeding Success estimates were divided by two, acknowledging the 50/50 sex ratio at the end of parental care [77]. Year 2007 was unique because only a subset of banded breeders were monitored. For the population projection, we estimated Annual Breeding Success in 2007 from a linear regression predicting Annual Breeding Success on the logit scale by annual survival on the logit scale (female age for both measures standardized at 10; β = -1.57, SE = 0.41, *P* = 0.001, adjusted *R*^*2*^ = 0.44). Second, we modeled juvenile survival from independence until recruitment (as a dichotomous variable, independent of the age at which an individual returned) predicted by maternal age (quadratic function) and year in a GLMM (binomial errors, logit link) with maternal identity as a random effect (N = 3,813 juveniles of unknown sex). We adjusted final estimates of annual juvenile survival to account for the 33% higher survival of males [57] and assumed a 50/50 sex ratio at independence. Variances of transformed age- and year-specific Annual Breeding Success estimates (divided by two) and juvenile survival estimates were adjusted using the delta method.

Data on juvenile survival are available for cohorts 1992–2008, but not for later cohorts because not all surviving members of those cohorts would have recruited at the time of our analysis. We exploited the positive relationship between annual mean breeding success (production of independent offspring) and annual mean juvenile survival to predict juvenile survival for these years using simple linear regression (β = 0.33, SE = 0.11, *P* = 0.01, adjusted *R*^*2*^ = 0.37). Data used in this analysis were the estimated (logit-scale) annual juvenile survival and Annual Breeding Success from the analyses described above (female age for both measures standardized at 10), and from all years 1992–2008 except 1997 and 1999. The 1997 *El Niño* event was excluded *a priori* based on the significance of the unusual environment for reproductive success. The 1999 season was a highly unusual year with almost no breeding success and was excluded after model checking indicated high leverage. Maternal age- and year-specific juvenile survival estimates were multiplied by age- and year-specific Annual Breeding Success estimates to give the F_*i*_.

Survival coefficients for each annual projection matrix, P_*i*_, for each age class *i* ≥ 4 were obtained using mark-recapture statistical methods that model survival while controlling imperfect detection probabilities [78]. Mark-recapture models were fit to encounter histories from the annual BRS over the period 1984–2013. These encounter histories were categorized into females banded as nestlings (N = 2,519) and females banded as adults (N = 813) by a grouping variable (“G”) identifying age-at-banding (during the hatch year, or as an adult). Encounter histories of all females who recruit as adults were set to begin at age 4. All birds banded as adults are assigned age 4 in their year of banding; thus, their encounter history began in the year of banding. For birds banded as nestlings, a handful appear in the BRS before age 4. Preliminary analysis indicated that survival estimates were essentially one for ages 2–3 and so these first appearances were not included in encounter histories.

Our most general model of survival probability, *Φ*, and recapture probability, *p*, allowed *Φ* and *p* to vary additively by breeding season and by age (factor, levels 4–21 and 22+). Age interacted with G whenever age appeared in any model, allowing distinct age-survival relationships for known-age individuals and those banded as adults. Year did not interact with age or group, constraining age-specific patterns to take the same form across all years. With both *Φ* and *p* dependent on time, survival across the final survival interval and recapture in the final BRS will be confounded, so our projection did not use *Φ* or *p* from the final period/recapture occasion (2013). Our mark-recapture model assumes that marks are not missed or lost, that the survey interval (~7 days) is short relative to the survival interval (1 year), and that *Φ* and *p* are homogeneous among individuals in the same group, age class, and year, with the added constraint that the relative differences between age-classes are constant through time. Leg bands are easy to detect and loss rates are extremely low in this population [55]. We examined the goodness-of-fit of our general model by estimating the overdispersion parameter, *ĉ*, using the median *ĉ* approach [79] run in Program MARK [80]. The estimate of *ĉ* (1.18, SE = 0.006) indicated adequate fit.

The most general model provided age- and year-specific survival estimates used to parameterize the annual projection matrices. To statistically evaluate an effect of Fish Phase on adult female survival we performed an AICc-based comparison of a model set including candidates constraining *Φ* to be equivalent either within Fish Phases (see below) or across all breeding seasons (S5 Table). Our model set evaluated only two alternative models for *p*: (1) *p* predicted by breeding season, and (2) *p* predicted by breeding season plus group (banding class) interacting with age. Candidate models for *Φ* included two different “Period” parameters. First, we included Period as a three-level factor with all Sardine Phase years (1984–1996), the 1997 *El Niño*, and all Flying Fish Phase years (1998–2013) as individual factor levels. Then, we modeled a similar effect, labelled “Period2”, but with the Sardine Phase broken into pre- and post-1992 sections, allowing direct comparison of Sardine Phase survival paired with breeding data and systematic diet sampling (available from 1992 onwards) with Sardine Phase survival from the previous 7 years. Demographic variation among years is large in our population. Thus, we expect the top model (evaluated by AICc comparison) to be fully time-dependent. We evaluated statistical support for the Period/Period2 effect in three ways: (1) by comparing the difference in AICc between the best performing (lowest AICc) model containing Period or Period2 and an otherwise-identical model that held survival constant across time, (2) by performing a likelihood ratio test between these two models, testing the effect of Period/Period2 [65], and (3) by considering the span of the 95% CI on the relevant coefficients. Maximum likelihood estimates of demographic parameters for each model were calculated using Program MARK and the RMark library [81]. These demographic parameters were related to predictors of interest using a logit link function.

The deterministic population growth rate (λ) for each annual projection matrix was calculated as the dominant eigenvalue [76]. Monte Carlo simulation was used to estimate 95% confidence intervals around each population growth rate. First, demographic parameters for each annual projection matrix were sampled randomly from beta distributions with mean and variance equal to the corresponding demographic parameter estimates to create 1000 projection matrices. The 2.5% and 97.5% quantiles of the resulting distribution of λs (after bias-correction to set the distribution mean to the exact asymptotic λ) was used to calculate the CIs for each λ. Deterministic λ for any given annual projection matrix shows the rate of population growth if the conditions experienced in that year were maintained indefinitely. To evaluate the cumulative effect of Fish Phase on all vital rates simultaneously, we used the annual matrix models associated with each Fish Phase to generate Fish Phase-specific stochastic λ values. Fish Phase-specific stochastic λ represents a long-term expectation of the population’s growth rate, given the finite set of vital rates (contained in the annual projection matrices) associated with each Fish Phase. This approach preserves the diversity of vital rates observed within each Fish Phase and the effect of such environmental variability on estimates of long-term population growth rate. Stochastic λ for each Fish Phase was calculated *via* simulation, by drawing independent and identically distributed sequences of annual matrices for each Fish Phase and estimating stochastic λ as the long-term average growth rate across 10,000 year-long intervals. Deterministic λs showed no evidence of between-year autocorrelation (*r* = 0.33, df = 18, *P* = 0.15), supporting our treatment of each breeding season as an independent environment. Simulations and approximate 95% CI on stochastic λs were calculated using R Package popbio [82], following ref. [76].

Deterministic population growth rate (λ) values for the annual projection matrices were calculated as the dominant eigenvalues of each projection matrix [76] and Monte Carlo simulation was used to estimate 95% confidence intervals around these population growth rates. We independently parameterized 1000 projection matrices for each annual projection matrix by randomly sampling demographic parameters (age class- and year-specific Annual Breeding Success, juvenile survival, and adult survival) from beta distributions with mean and variance equal to the corresponding demographic parameter estimate and used the 2.5% and 97.5% quantiles of the resulting distribution of λs (after bias-correction to set the distribution mean to the exact deterministic λ) to calculate the CIs. The deterministic λ for any given annual projection matrix illustrates the consequences of indefinitely maintaining the conditions experienced in that year; however, collectively, the deterministic λs do not illuminate the effect of diet. We evaluated the time series of deterministic λs for evidence of between-year autocorrelation, and finding none (*r* = 0.33, df = 18, *P* = 0.15), we used the annual matrix models associated with each Fish Phase to generate Fish Phase-specific stochastic λ values. Fish Phase-specific stochastic λ represent a long-term expectation of the population’s growth rate, given the finite set of vital rates (contained in the annual projection matrices) associated with each Fish Phase. This approach thus accounts for the diversity of vital rates observed within each Fish Phase and the effect of such environmental variability on long-term population growth rate estimates. Stochastic λ for each Fish Phase was calculated *via* simulation, by drawing independent and identically distributed sequences of annual matrices for each Fish Phase and estimating stochastic λ as the long-term average growth rate across 10,000 year-long intervals. Simulations and approximate 95% CI on stochastic λs were calculated using R Package popbio [82], following ref. [76].

All methods were approved by the Wake Forest University Institutional Animal Care and Use Committee (IACUC; protocol no. A11-051 and earlier).

## Results

### Food limitation

During the Flying Fish Phase, Nazca boobies experienced lower energy density in their diet [39,40], smaller prey [34], and lower foraging success (Fig 1b), implying stricter food limitation than during the Sardine Phase. Nestling growth gave strong evidence of this limitation after the switch from the Sardine Phase to the Flying Fish Phase. Nazca boobies raise a single offspring per year [83], and the typical time between that offspring’s hatching and acquisition of juvenile plumage [56,57] was 8 days longer during the Flying Fish Phase than during the Sardine Phase (linear mixed model β = -8.31, 95% CI = [-13.4, -3.1]; likelihood ratio test, *Χ*^2^ = 8.87, df = 1, *P* = 0.003; S6 Table, details in S1 Methods). Natural selection favors fast growth in this species because fast-growing nestlings have higher survival after becoming independent [57]; slow growth indicates a nutritional deficit. This lengthening of the nestling period implies at least a 7% reduction in survival of newly independent offspring [57] during the Flying Fish Phase.

### Breeding success

**Fig 3:**
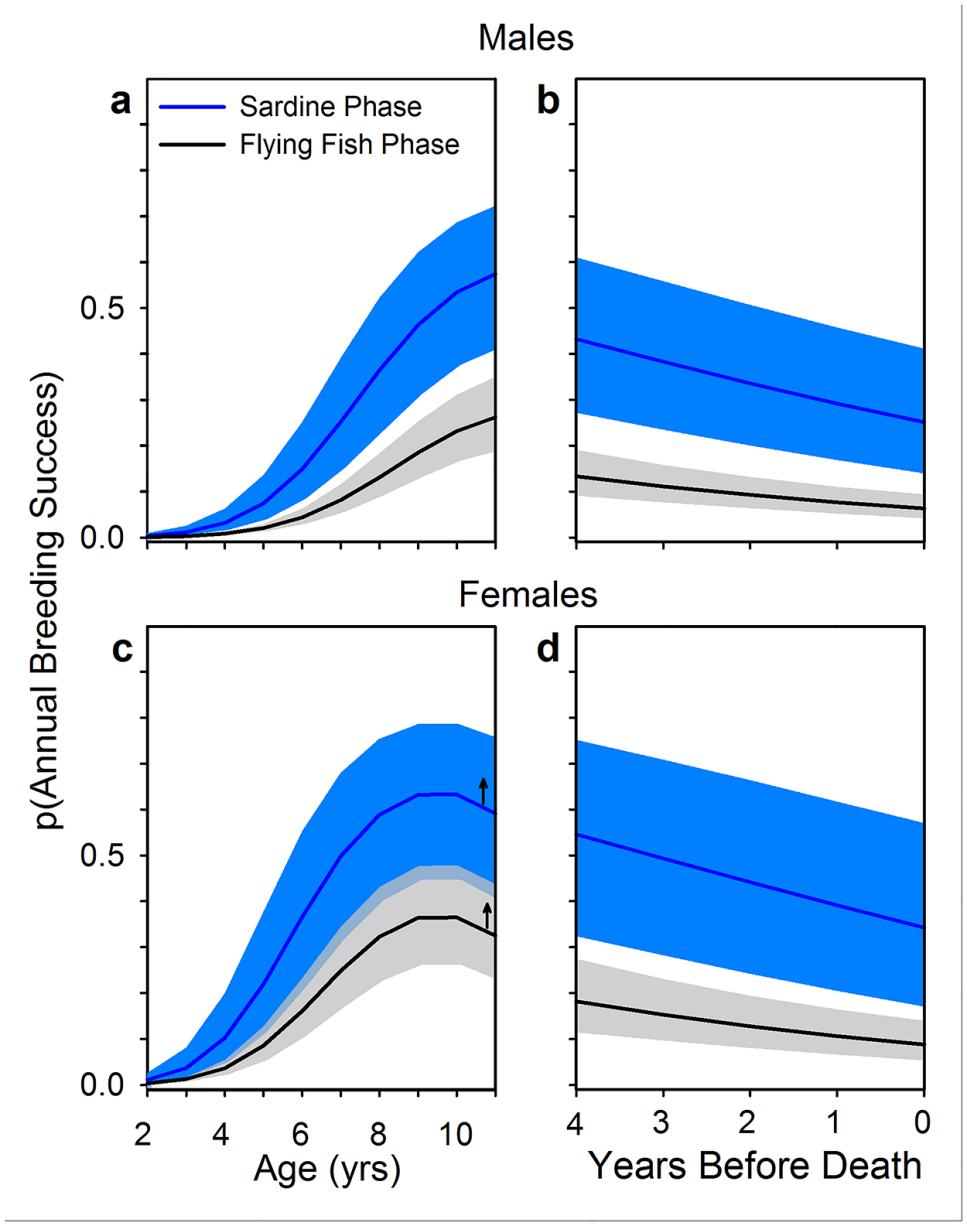
Annual Breeding Success by Fish Phase. Annual Breeding Success is the probability of producing an independent offspring if alive in that year. Results shown for young and middle-aged males (a) and females (c), and for presumed old males (b) and females (d). Curves (± 95% CI) are predictions from generalized linear mixed models with a binomial error structure, controlling annual SSTA variability. Data come from all 19 years of the study, including years without other breeding details (negative slope for females aged 10–11 is an artifact of fitting a quadratic function to an age series that becomes level at age 8–9 yrs [see S5 Fig], as indicated by arrows). Full model results given in S1–S4, S7 and S8 Tables.

**Fig 4:**
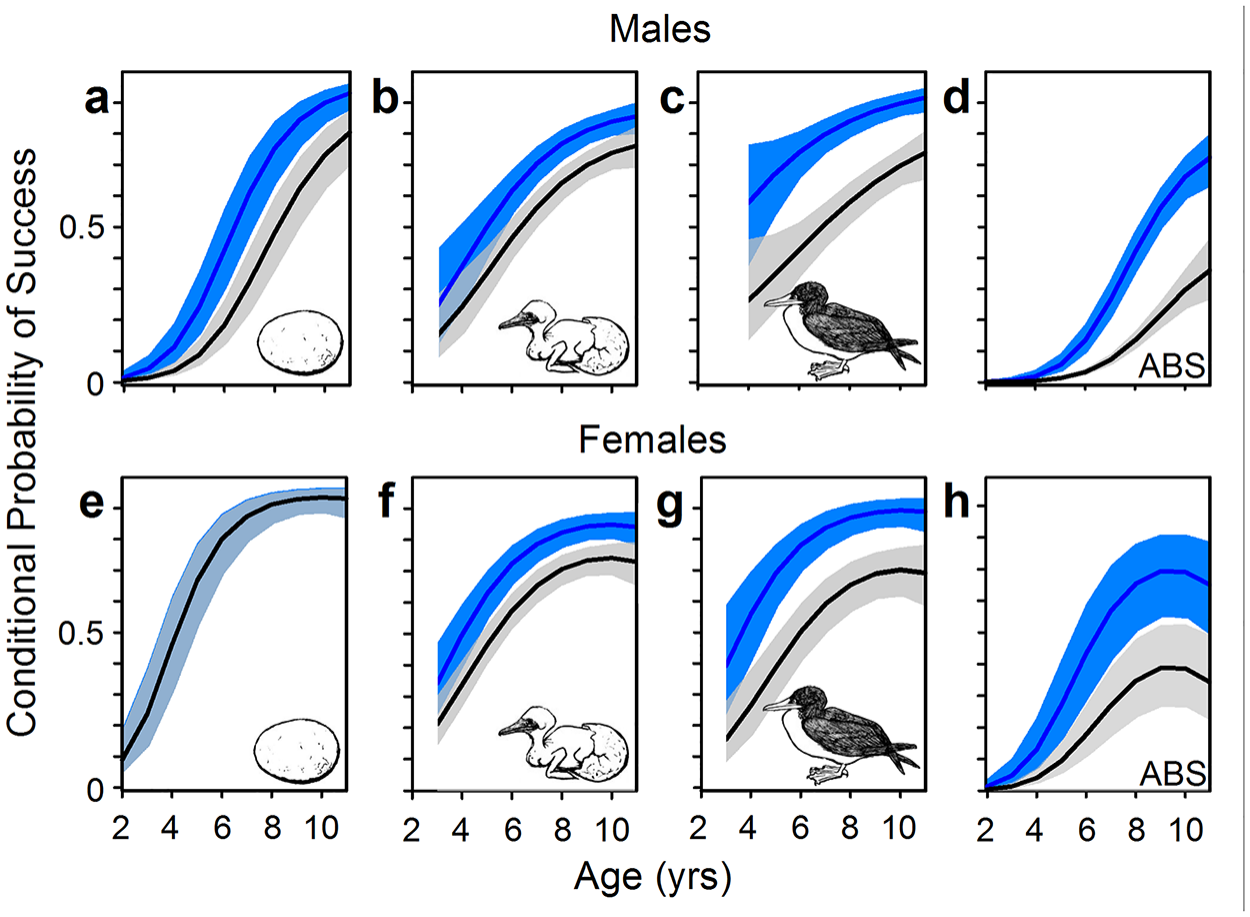
Component stages of Annual Breeding Success of young and middle-aged adults by Fish Phase. Predictions for Sardine Phase (blue) and Flying Fish Phase (gray) shown as in Fig 3. Data are derived only from the 12 yrs of the study with detailed histories on the components of each breeding attempt. See Methods for years involved in each stage. Conditional probabilities for different stages are p(initiate a clutch | alive) (panels a, e), p(hatch an egg | clutch was initiated) (panels b, f), p(produce independent offspring | hatch) (panels c, g), and p(produce independent offspring | alive) for only the subset of years for which analysis of components was possible (panels d, h). Sample size varies by sex/response variable. Full model results given in S1, S2 and S7 Tables.

Under the nutritional deficit imposed by a flying fish diet, we predicted lower breeding success during the Flying Fish Phase than during the Sardine Phase. Nazca boobies begin to breed successfully at approximately age 4 yrs, improve breeding performance up to age 8, and decline in performance between ages 15 and 21 (S5 Fig). Controlling these age effects from early adulthood until the peak in breeding success in middle age, we found that Annual Breeding Success (probability of producing an independent offspring given that bird was alive in that year) declined by approximately 50% during the Flying Fish Phase for each sex (Fig 3a and 3c). Females’ poor performance during the Flying Fish Phase was due primarily to failure to hatch eggs and to raise hatchlings to independence, while the probability of initiating a breeding attempt (laying eggs) was similar in the two Fish Phases (Fig 4, S1 and S7 Tables). Young and middle-aged males showed similar deficits, as well as fewer initiations of breeding, in the Flying Fish Phase (Fig 4, S2 and S7 Tables). The sigmoid patterns of early life improvement were similar across Fish Phases, aside from markedly depressed performance during the Flying Fish Phase (all Age x Fish Phase interaction coefficients, with data on a logit scale, were close to zero and excluded from top models). Our long-term study has revealed no other environmental effects (e.g., predation, disease, anthropogenic effects) that can help explain the Fish Phase effect on Nazca booby demography. The higher elevation of females’ values than males’ for Annual Breeding Success (Fig 3) and clutch initiation (Fig 4) are both products of the mating system of this population: both sexes provide extensive parental care throughout the breeding attempt, but a strongly male-biased adult sex ratio excludes approximately 1/3 of males from the breeding pool in a given year [66].

**Fig 5:**
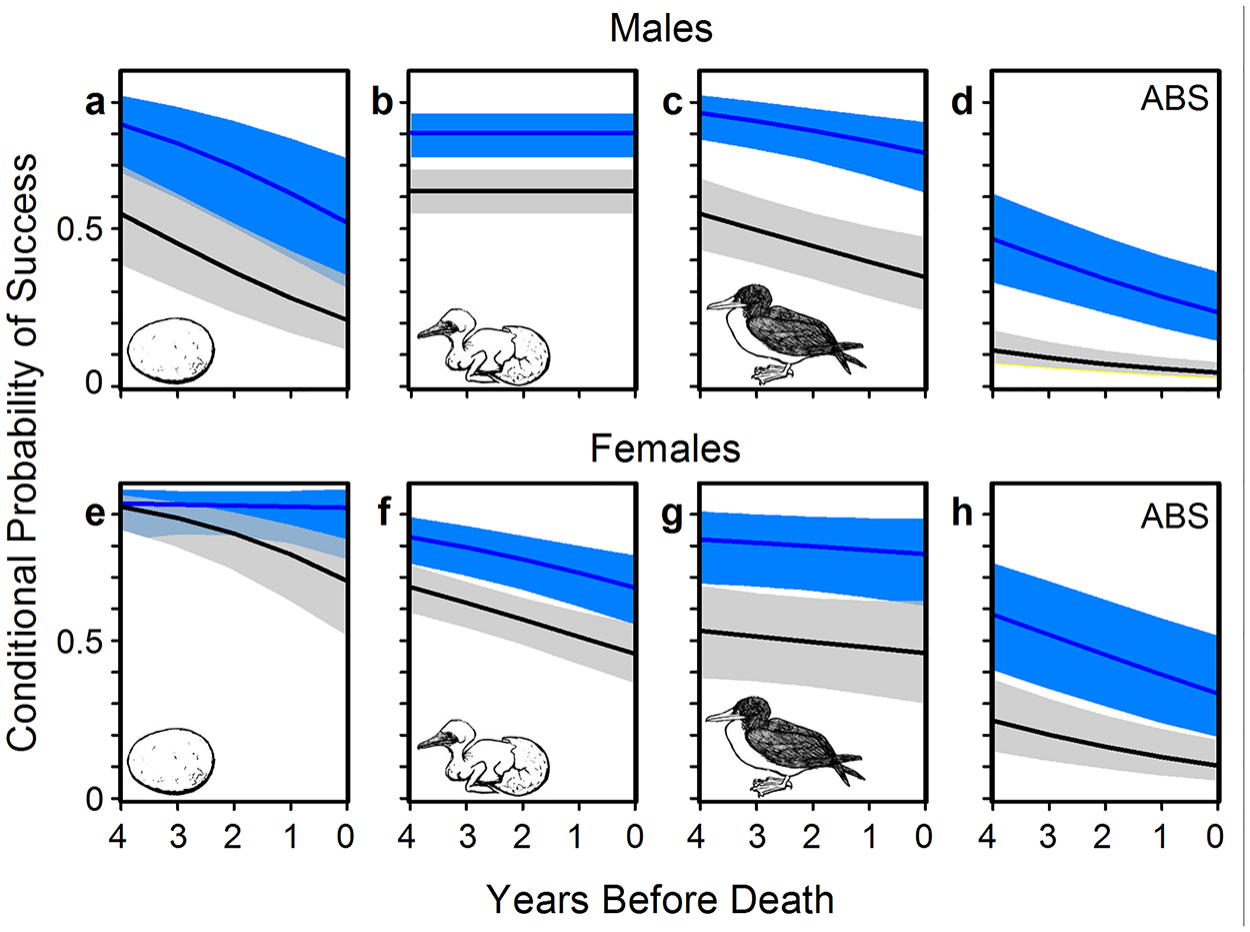
Component stages of Annual Breeding Success of presumed old adults by Fish Phase. Predictions for Sardine Phase (blue) and Flying Fish Phase (gray) shown as in Fig 3. Data are derived only from the 12 yrs of the study with detailed histories on the components of each breeding attempt. See Methods for years involved in each stage. Conditional probabilities for different stages are p(initiate a clutch | alive) (panels a, e), p(hatch an egg | clutch was initiated) (panels b, f), p(produce independent offspring | hatch) (panels c, g), and p(produce independent offspring | alive) for only the subset of years for which analysis of components was possible (panels d, h). Sample size varies by sex/response variable. Full model results given in S3, S4 and S8 Tables.

Comparing breeding success across Fish Phase for adults in senescent decline, to complete analysis across the lifespan, relied on YBD, a proxy for chronological age [63]. As with young and middle-aged adults, Fish Phase affected Annual Breeding Success and its component stages similarly across the range of YBD (Fig 3b and 3d; interactions between YBD and Fish Phase were poorly supported for both sexes for Annual Breeding Success and all component stages; S3, S4 and S8 Tables). Birds in the five years preceding death showed dramatically lower breeding success during the Flying Fish Phase at almost all stages of breeding, although the effect for females initiating breeding was weak (Fig 5). Note that the evidence of senescent decline in Fig 5 is conservative because YBD confounds the flat or shallow performance trajectories of birds dying in late middle age with the steeper trajectories of truly old individuals (see S5 Fig).

### Population growth rate and Fish Phase

We parameterized the models of population growth rate (λ) with our breeding data and with age-specific data on annual adult survival. Candidate models for adult female survival that grouped time into a three-level factor (“Period”: Sardine Phase 1984–1996, the 1997 *El Niño*, and Flying Fish Phase 1998–2013) ranked higher than models with time-independent survival (S5 Table; LRT comparing models with and without Period: *Χ*^*2*^ = 192, df = 2, *P* << 0.001; see S1 Methods). Grouping time into a four-level factor as above, except Sardine Phase was split into 1984–1991 (when diet data were sparse) and 1992–1996 (consistent diet of sardine), was less parsimonious (S5 Table). This result validates the comparison between Sardine Phase and Flying Fish Phase using Period as a three-level factor, ignoring the sparseness of the diet data from 1984 to 1991. Costs of reproduction may have been reduced by the poor breeding success under a flying fish diet: survival was ~4% higher in the Flying Fish Phase than in the Sardine Phase (S2 Fig; 95% CI for increased survival during the Flying Fish Phase relative to the Sardine Phase does not span zero, β = 0.55, 95%CI = [0.39, 0.70]). Inter-annual variation in annual survival probability within each Fish Phase was important: the model differentiating each breeding season 1984–2013 in a multi-level factor best explained variation in the data (S5 Table). Annual survival was typically ≥ 0.90 (27/28 seasons at age 10, decreasing with age in known-age birds) and annual recapture probabilities were ≥ 0.90 for most years (24/28 seasons at age 10, increasing and then decreasing with age in known-age birds).

**Fig 6:**
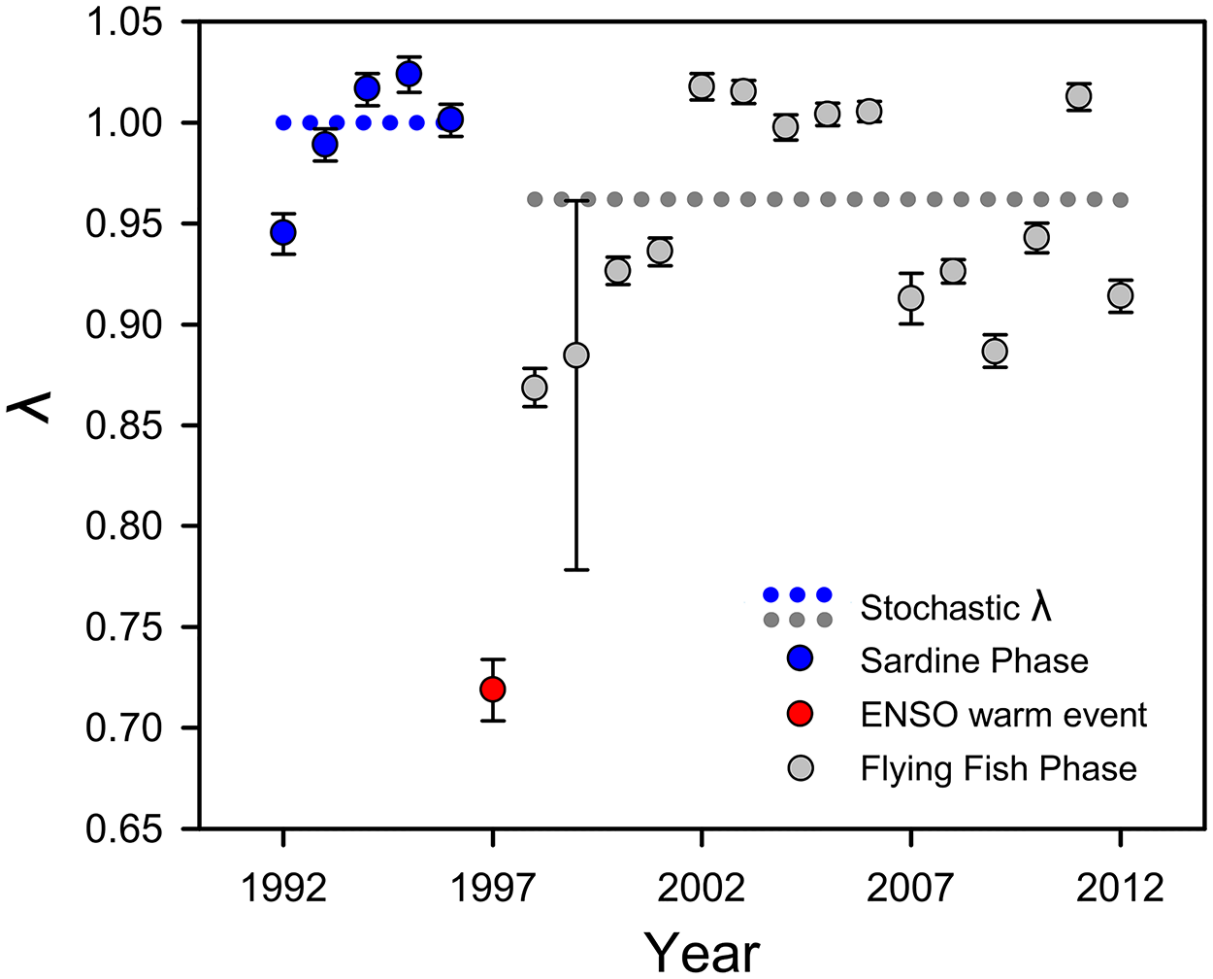
Deterministic annual λ (population growth rate) values from age-classified, single-sex (female) population projection models for Nazca boobies. λ > 1 signifies a growing population. Brackets show 95% CIs. Interval is particularly large for 1999 because of almost complete breeding failure and nearly complete survival for most age classes, so that the rate of population shrinkage depends disproportionately on survival in the final age class (age 22+), a value predicted with low precision because sample size declines with age. Stochastic λ values specific to each fish phase shown with dotted lines.

Stochastic λ (the long-term average growth rate across 10,000 simulated projection intervals) for the Flying Fish Phase was markedly less than 1 (0.963), with a 95% confidence interval [0.962, 0.964] excluding 1 (Fig 6). In contrast, stochastic λ = 1.000 estimated from the last five years of the Sardine Phase (95% CI = [0.999, 1.000]; Fig 6). The stochastic λ for the Flying Fish Phase is robust: it is based on 15 years of data encompassing wide variation in fertility and survival (S2 and S4 Figs), which yield corresponding annual variation in λ due to a source of variation that has not been accounted for. A population decline is projected during the Flying Fish Phase. To avoid a stochastic λ less than 1 during the Flying Fish Phase, a very large increase (~55%) in one of the demographic rates underlying fertility (juvenile survival or Annual Breeding Success) is necessary, applied across all years and all relevant demographic parameters.

## Discussion

When Nazca boobies lost Pacific sardines from their diet their expected breeding success fell dramatically (Fig 3). Adult males and females of all ages had much lower success hatching eggs and raising nestlings during the Flying Fish Phase (controlling individual identity and other variables; Figs 4 and 5). Additionally, males of all ages had lower success initiating breeding during the Flying Fish Phase (Figs 4 and 5). As a direct consequence, and despite higher adult survival, during the Flying Fish Phase the stochastic population growth rate λ was less than 1, forecasting a shrinking population. Nazca boobies showed more diet flexibility than blue-footed boobies did during the Sardine Phase [34] and were able to adjust their foraging enough to raise some offspring during the Flying Fish Phase, unlike the sardine specialist blue-footed boobies [52]. However, the Nazca boobies’ foraging plasticity and complementary shifts in breeding and adult survival were insufficient to cope with the loss of sardines. Neither booby species can maintain a stable population under the conditions of the Flying Fish Phase at our Galápagos site. With a lipid content of < 1% [84], flying fish are effectively “junk food” (*sensu* [85,86]) compared to sardines (lipid content 8%; [87]).

This result provides rare evidence that ocean warming will affect a tropical marine vertebrate: the contemporary effect of a long-term qualitative change in diet on vital rates forecasts the demographic trajectory of this predator if, as seems likely, the predicted acceleration in ocean warming in the ETP makes sardines functionally absent from the Nazca booby’s present foraging range. Pelagic seabirds are well known for extreme mobility, yet extending the foraging range from the current breeding site is not a remedy. During the Sardine Phase few trips exceeded a single daylight period and most birds foraged ~65km from the colony [88], but comparable data from the Flying Fish Phase are already several times those values [48]. If the current link between diet and breeding applies in the future, and sardines are unavailable, then this Nazca booby population will shrink dramatically in a persistent Flying Fish Phase. Detecting changes in actual counts of birds is a task for the future: annual estimates of λ from our matrix models show that shrinkage of the countable adult population should only now begin to be detectable (data not shown), due to this species' extended pre-breeding period, when they are at sea and not available to us to count, and to a bottom-heavy age structure for much of the Flying Fish Phase to date.

Large-scale range contractions and multi-decadal oscillation in population sizes of the Pacific sardine are well known on the continental margin around most of the Pacific, where fishery data provide long time-series of population abundance [89]. The historical record of the Galápagos population of sardines begins in the early 1980s (Galápagos lacks an industrial sardine fishery), providing too short a time span to evaluate the idea that sardine availability to Galápagos boobies oscillates. On one hand, the unusual “oceanic” location of Pacific sardines in Galápagos, distant from the important influences of the continental shelf and shoreline [42], may remove the drivers of the oscillation. If so, the Flying Fish Phase may be a novel foraging environment for which Nazca boobies are poorly prepared. On the other hand, the population of Pacific sardines in the nearby Peruvian Upwelling was large from the early 1970s until the mid-1990s, and nearly absent since then [89,90], roughly congruent with the pattern in Galápagos (Fig 1). In that case, the conditions of the Flying Fish Phase would occur regularly in the evolutionary history of this population and would not be novel. Pacific sardines are strong swimmers, with individuals ranging widely within their lifespans to track their habitat requirements, although typically on the pelagic edge of continental shelves [42]. Active movement or passive transport in the northwest-flowing Humboldt Current from the Peruvian Upwelling to Galápagos has not been demonstrated but appears to be possible. Any such source-sink or migratory connection between the sardines in the Peruvian Upwelling and the Galápagos population could explain the recent diet shift of Galápagos marine predators.

The availability of Pacific sardines to Nazca boobies and other top predators in Galápagos ([41,52]; Fig 1) during the Sardine Phase may be puzzling, given the frequency with which SST exceeded the sardines’ thermal tolerances in Fig 2b. The coarse 2° x 2° spatial scale of that analysis admits the possibility of small thermal refuges that could attract sardines during warm periods. Consistent with this idea, radio-tracking in the 1980s showed that most foraging of both Nazca and blue-footed boobies occurred at two hotspots above seamounts that might have deflected cool subsurface water to the surface [88]. Since these booby species were capturing mostly Pacific sardines at that time [34], their foraging distribution implies an abundance of sardines over the seamounts. A variety of sub-surface predators also concentrated foraging activity at these sites [88]. Galápagos waters probably provided a marginal and patchy environment for Pacific sardines during the Sardine Phase of our study, yet sardines were effectively absent from the Nazca boobies’ foraging range during the Flying Fish Phase without any meaningful change in SST (Fig 2). We infer from this that population dynamics on the continental margin, with a population crash around the time that our Sardine Phase ended [89], combined with transport or active movement on the Humboldt Current to Galápagos, provides the best explanation for sardine availability in Galápagos. Sardines in the Humboldt Current Ecosystem associate with a temperature range of 9–25°C [91], suggesting similar temperature preference and tolerance to those recorded for temperate-origin, warm-adapted sardines from the California Current Ecosystem used to measure UILT [44]. Regional warming of 4.5°C in Galápagos due to ocean warming expected within 100 years will move SST above the UILT and maximum spawning temperature of warm-acclimated temperate-origin Pacific sardines in all 12 months of an average year, and in all but 3–4 months of unusually cool years (Fig 1c). Although we cannot rule out the persistence of micro-scale thermal refugia within the archipelago under the predicted warming, these results suggest that acceptable habitat will become extremely rare, both temporally and spatially.

The oscillation of sardines on the continental margin is approximately anti-phase with that of anchovies (*Engraulis* spp.; [89]). Predators there can switch between sardines and anchovies, depending on availability, and obtain high quality schooling prey in either case. Our diet samples never contained anchovies, and predators of sardines in Galápagos evidently struggle when sardines are rare, lacking a comparable replacement. If the Galápagos sardines are an oscillating population, then the cycle over the continental shelf suggests that the prey population will rebound in roughly 5 to 10 years [89,92]. But regardless of past and present population dynamics of Pacific sardines in Galápagos, the considerations summarized in Fig 2 indicate that in the longer term sardines will be functionally absent from the diet.

These results forecast poor conditions at this population’s current location due to the indirect trophic effect of the ocean warming component of climate change. Nazca boobies show remarkable fidelity to that location: virtually all individuals breed within a few hundred meters of their natal nest [55]. Nonetheless, exceptional individuals do settle elsewhere [55], so a range shift [4] appears to be possible. A continental or fully marine animal might have the option of shifting its range across relatively continuous habitat to meet its critical ecological requirements. In contrast, seabirds in much of the world’s oceans live in a fundamentally discontinuous habitat, punctuated by the location of breeding islands. In the case of an archipelago like Galápagos, our study population on the southernmost Galápagos island might move to a northern island, but SST is even higher to the north, following a consistent gradient influenced by the Humboldt Current in the south [93]. They might move to the western margin of Galápagos where the cool upwelling of the Equatorial Countercurrent [93] could provide a refuge of limited extent for a point (probably unstable) population of sardines and a remnant population of Nazca boobies. Outside Galápagos, the islands within 2000 km include only one (Cocos) that is not either already occupied by Nazca boobies (Malpelo, La Plata, Lobos de Tierra, and Lobos de Afuera) or already occupied by congeneric habitat specialists that are probably competitive superiors in that area (guano islands in the Peruvian Upwelling). Our study population of Nazca boobies is the largest of several in Galápagos, representing approximately 60% of the archipelago’s population [55]. Only one other significant breeding site exists, on Isla Malpelo (4° 00’N 81° 36’W), 1000 km to the northeast, supporting the world’s largest [94], but poorly documented, Nazca booby colony. While SST around Malpelo is predicted to rise ~4°C [32] and will also be unsuitable for Pacific sardines, it cannot be excluded as a suitable future breeding site without knowing more about the local diet and its links to breeding success. Nonetheless, range reduction to a single significant colony at Malpelo would represent a consequential change in conservation status.

The example of Nazca boobies illustrates the special difficulties that island-breeding, ocean-feeding vertebrates will face in a rapidly changing climate. Nonetheless, detecting and predicting the effects of climate change on these animals will generally be challenging, despite their relative conspicuousness [31]. Indeed, a long-term study of Nazca boobies limited to only one Fish Phase could not have detected the dramatic demographic effect of the disappearance of sardines. The projection of this population’s decline under ocean warming, despite little contemporary change in the ETP, is clarified by several circumstances: Nazca boobies are top predators (higher trophic levels do not impinge on their population dynamics), their diet composition is simple, and loss of their sardine prey is catastrophic. Abiotic, especially thermal, effects of climate change on populations are pervasive and well-documented, but biotic effects of trophic and other biological interactions are acknowledged less often [28]. Biotic effects similar to that of sardines on Nazca boobies are probable for other species pairs whose separate niches include different habitat types that are themselves affected differently by climate change (for example, polar bears (*Ursus maritimus*) and their marine prey [95]. Our results may also forecast, more generally, population trends of many organisms with a close ecological tie to another, but with contrasting capacities for range shift as the climate changes.

## Supporting information

S1 Methods (DOCX)

S1 Fig Recent SST + 4.5°C and sardine spawning temperature east of the Nazca booby foraging range. (a) Foraging envelope [48] (semicircle) of breeding Nazca boobies from Isla Española, Galápagos. Most present-day trips are within the area of the semicircle. Dotted line to the east shows the continental shelf break (1000 m isobath), roughly indicating the distribution of continental Pacific sardines, a probable source population for Galápagos. (b) Recent temporal variation (blue lines) in SST in each of 12 2° x 2° blocks east of the present-day foraging range of Nazca boobies. Solid horizontal line shows upper limit of spawning SST range of Pacific sardines (25°C; [45–47]). Dashed horizontal line shows upper incipient lethal limit for warm-acclimated temperate-origin Pacific sardines (25.6°C; [44]). (c) Recent SST from (a) with 4.5°C warming expected within 100 years. Orange pts (± 2 S.D.) show expected temperature averaged across all 12 blocks and across all years (1982–2016) by month. Gray lines show temperatures averaged across all years, by month, for each individual block. Monthly SST values for each 2° x 2° block were downloaded on 12 May 2016 from http://iridl.ldeo.columbia.edu/SOURCES/.NOAA/.NCDC/.ERSST/.version3b/.sst/.
(TIF)

S2 Fig Annual probabilities of adult female survival (a, b) and annual probabilities of raising a female offspring to independence, given that the mother is alive, (c, d) used to construct annual population projection matrices. (a) Age-specific annual survival probabilities of adult females (N = 3,332 individuals) for each of the years 1985–2012 estimated with a mark-recapture model controlling encounter probabilities <1; year and age fit additively as multi-level factors (ages 4–21, 22+). (b) Temporal sequence of annual survival probabilities holding age constant at 10 yrs; brackets show 95% CIs. (c) Age-specific Annual Breeding Success (N = 14,640 breeding records) for 1992–2006, 2008–2012 from a binomial GLMM (logit link); year and age fit additively as multi-level factors (ages 2–21, 22+). In 2007, value was estimated using annual survival (see S1 Methods). (d) Temporal sequence of Annual Breeding Success holding age constant at 10 yrs; brackets show 95% CIs.
(TIF)

S3 Fig Annual patterns of “age-” specific recapture (a) and survival (c) probabilities for female Nazca boobies banded as adults and for known-age females (b, d). Estimated age assigned as 4 in the year of banding for individuals banded as adults. Survival and recapture probabilities from the top mark-recapture model allowing both probabilities to vary by year and by banding class (as adult or as young of the year) interacting with age (multi-level factor, levels 4–21, 22+). Values for individual years 1985–2012 are in grey, with the median age-specific trajectory marked by a thick black line.
(TIF)

S4 Fig Annual probabilities of juvenile survival for female Nazca booby offspring by mother’s age (a) and in temporal sequence (b) used to construct annual population projection matrices. (a) Curves are predictions for each year from a GLMM (binomial errors, logit link) modeling offspring survival from independence to recruitment by mother’s age (continuous; linear and quadratic predictors) and year (factor, 1992–1996, 1998, 2000–2008), N = 3,813. (b) Temporal sequence of annual values holding a mother’s age constant at 10 yrs. Checkered points (2009–2012) mark years too recent for cohort members to have fully recruited; for these years, annual rate of juvenile survival was predicted from its positive relationship with Annual Breeding Success. Brackets show 95% CIs. Offspring sex was unknown for some cohorts, so the sexes were combined for modeling and all values were adjusted for the 33% lower survivorship of female offspring compared to male [51]. Few offspring reached independence in 1997 (N = 18) and 1999 (N = 10) and none survived the juvenile period, preventing inclusion of these years in the GLMM.
(TIF)

S5 Fig Annual Breeding Success by Fish Phase for known-age Nazca boobies. Breeding records of (a) males (N = 19,635 breeding records) and (b) females (N = 14,302 breeding records) during five seasons of Sardine Phase (1992–1996) and 14 seasons of Flying Fish Phase (1998–2012). Predicted mean Annual Breeding Success (95% CIs) by age (fit as a multi-level factor), Fish Phase, and their interaction, with year and identity random effects from sex-specific GLMMs (binomial errors, logit link). Sardine Phase age classes truncated at 12 because the diet switch occurred before old adults were produced by earlier banding of young of the year.
(TIF)

S1 Table Model selection using AICc to rank GLMMs (binomial errors, logit link) explaining variation in Annual Breeding Success and sequential reproductive stages in young and middle-aged (≤ 11 yrs) female Nazca boobies. Models within ΔAICc of 2 are considered to be highly supported (in bold) unless they are more complex, nested versions of the top model. Such a model is penalized only 2 AIC units for each additional term and appears to be well-supported despite little variance explained by the additional fixed effect(s). Models ΔAICc ≥ 7 from the top model for each response variable are not presented. FP: a dichotomous factor for Fish Phase; SSTA_AMJ_ and SSTA_DJF_: local sea surface temperature anomalies averaged across Apr-Jun and Dec-Feb, respectively; *El Niño*: a dichotomous factor marking the 1997–98 extreme ENSO warm event. Main effects (Age + FP) plus the interaction between Age and FP are written as “Age x FP”. All models included female identity and year as random effects. The number of parameters (*k*), small sample size-corrected AIC value (AICc), AICc difference from the top model (ΔAICc), and Akaike weights (*ω*_*i*_) are reported.
(DOCX)

S2 Table Results as in S1 Table, but for young and middle-aged male Nazca boobies. (DOCX)

S3 Table Model selection using AICc to rank GLMMs (binomial errors, logit link) explaining variation in Annual Breeding Success and sequential reproductive stages in presumed old female Nazca boobies. Models within ΔAICc of 2 are considered to be highly supported (in bold) unless they are more complex, nested versions of the top model. Such a model is penalized only 2 AIC units for each additional term and appears to be well-supported despite little variance explained by the additional fixed effect(s). Models ΔAICc ≥ 7 from the top model for each response variable are not presented. YBD: centered, continuous, years before death; FP: a dichotomous factor for Fish Phase; SSTA_AMJ_ and SSTA_DJF_: local sea surface temperature anomalies averaged across Apr-Jun and Dec-Feb, respectively. Main effects (YBD + FP) plus the interaction between YBD and FP are written as “YBD x FP”. All models included female identity and year as random effects. The number of parameters (*k*), small sample size-corrected AIC value (AICc), AICc difference from the top model (ΔAICc), and Akaike weights (*ω*_*i*_) are reported.
(DOCX)

S4 Table Results as in S3 Table, but for presumed old male Nazca boobies. (DOCX)

S5 Table Model selection results for a mark-recapture analysis of adult female survival (*Φ*) and recapture probabilities (*p*) using Program MARK. Predictors include Year: a multi-level factor; Age: a multi-level factor (4–21, 22+); G: group, either banded-as-adult or banded-as-chick; P: Period, a three-level factor grouping the Sardine Phase (1984–1996), the 1997 *El Niño*, and the Flying Fish Phase (1998–2013); an alternate version of P, P2, had four levels 1984–1991, 1992–1996, 1997, and 1998–2013, allowing a test for differences in survival between Sardine Phase years for which we have reproductive data and systematic diet sampling *vs*. earlier years. The number of parameters (*k*), small sample size-corrected AIC value (AICc), AICc difference from the top model (ΔAICc), and Akaike weights (*ω*_*i*_) are reported for the complete model set. Main effects (G + Age) plus the interaction between G and Age are written as “G x Age”.
(DOCX)

S6 Table Coefficient estimates from an LMM evaluating Nazca booby offspring growth rates (measured as offspring age at the 1% remaining down stage) by Fish Phase. FP: a dichotomous factor for Fish Phase; SSTA_AMJ_ and SSTA_DJF_: local sea surface temperature anomalies averaged across Apr-Jun and Dec-Feb, respectively. The variance for the random effects associated with each model are presented along with the intraclass correlation coefficient (ICC; the proportion of total variance not accounted for by fixed factors) in brackets.
(DOCX)

S7 Table Coefficient estimates for the GLMM (binomial errors, logit link) best explaining variation (lowest AICc value; the “top model”) in Annual Breeding Success and sequential reproductive stages in young/middle-aged (≤ 11) male and female Nazca boobies. Age: centered, continuous; FP: a dichotomous factor for Fish Phase; SSTA_AMJ_ and SSTA_DJF_: local sea surface temperature anomalies averaged across Apr-Jun and Dec-Feb, respectively; *El Niño*: a dichotomous factor marking the 1997–98 extreme ENSO warm event. SSTA_AMJ_ is not included as a predictor for traits expressed prior to April. The variance for the random effects associated with each model are presented along with the intraclass correlation coefficient (ICC; the proportion of total variance not accounted for by fixed factors) in brackets.
(DOCX)

S8 Table Coefficient estimates for the GLMM (binomial errors, logit link) best explaining variation (lowest AICc value; the “top model”) in Annual Breeding Success and sequential reproductive stages in presumed old male and female Nazca boobies. YBD: centered, continuous, years before death; FP: a dichotomous factor for Fish Phase; SSTA_AMJ_ and SSTA_DJF_: local sea surface temperature anomalies averaged across Apr-Jun and Dec-Feb, respectively. SSTA_AMJ_ is not included as a predictor for traits expressed prior to April. The variance for the random effects associated with each model are presented along with the intraclass correlation coefficient (ICC; the proportion of total variance not accounted for by fixed factors) in brackets.
(DOCX)
