# Supplementary material for: Decadal-scale variation in diet forecasts persistently poor breeding under ocean warming in a tropical seabird
Source: PLoS One. 2017 Aug 23;12(8):e0182545. doi: 10.1371/journal.pone.0182545 (PMC5568137; doi:10.1371/journal.pone.0182545)
Supplement: S1 Methods — (DOCX) [file pone.0182545.s001.docx]

**Decadal-scale variation in diet forecasts persistently poor breeding under ocean warming in a tropical seabird**

Emily M. Tompkins, Howard M. Townsend, and David J. Anderson

**S1 Methods**

**Data collection: Diet sampling**

For years in which we collected diet data (Fig. 2), samples were collected systematically by regurgitation from 20 adults (split roughly evenly between the sexes) at the beginning of each month. These systematic samples came from randomly selected adults returning from a foraging trip between 1700-1900 h. We recorded whether the bird produced a sample and identified any prey produced (usually to family, although sardines to species), and in most cases induced the captured bird to consume the sample again. Propensity to regurgitate a sample varied across the annual cycle, and we standardized the sampling period by using only data from samples in months common to all years (January-April; Fig. 2b). Birds sometimes regurgitated spontaneously on other occasions, and we combined these with samples collected systematically for aggregated annual summaries of number and type (Fig. 2a).

**Data collection: Arrival date**

Until 2001, encompassing all of our years in the Sardine Phase, we arrived at the colony to begin monitoring late enough (mean arrival date 12 November) to miss some early clutches: an average of 52% of clutches were already established during that period, with almost all still at the egg stage and most of those initiated within 15 days before our arrival. This late arrival does not affect assignment of Annual Breeding Success (and therefore does not affect the population projection analysis), or assignment of production of an independent offspring given that a nestling was hatched because no bias exists in detection of offspring at their age of independence (reached late in the season, when we were always present). Undetected failed initiations in the Sardine Phase may depress our estimate of the probability of initiating a nest (reduce the difference between phases) and inflate our estimate of the probability of hatching at least one nestling (increase the difference between phases) in the Sardine Phase relative to the Flying Fish Phase. Using data from the Flying Fish Phase (1998 and later), unaffected by this issue, we evaluated the potential effects of biased sampling by ignoring all clutches that failed before 12 November and re-running models describing the probability of initiating a clutch and the probability of producing at least one hatchling for young/middle-aged and presumed-old birds of both sexes. This exercise showed that the proportion of clutches laid before, *and failing* *before*, 12 November varied by age/sex/trait combination but were a very small percentage of the nests initiated during that period (maximum = 9%, minimum = 3%). Failure rates overall were higher during the Flying Fish Phase (Figs. 3, 4), suggesting that few failed clutches would have been missed before 2001, when the crew arrived late. The magnitude of any bias is small: if we remove all early clutches from the Flying Fish Phase years to standardize the data collection period across Fish Phase, the absolute change in the probability of initiating a nest during the Flying Fish Phase (see Figs. 3, 4) was a reduction of 0.02-0.04 for all age/sex/trait combinations (see Figs. 3, 4 for context). This magnitude was insignificant compared to the confidence intervals surrounding predicted values (see Figs. 3c, h and Figs. 4c, h for CIs) and any actual bias would enhance, not reduce, the importance of Fish Phase in our results. The magnitude of absolute change was even less for the probability of producing a hatchling, although in the direction of increasing hatching success in the Flying Fish Phase, so that any actual bias would reduce the importance of Fish Phase for the probability of hatching offspring given a clutch’s initiation. In summary, the variability of our arrival date at the colony induced little, if any, bias in evaluation of Fish Phase or other effects, because breeding failure before 12 November is rare.

**Statistical analysis: Food limitation**

To address the possibility of food limitation during the Flying Fish Phase, we evaluated the number of days between hatching and acquisition of juvenile plumage (the 1% down stage) using data on 4,075 offspring raised during 1992-1996 in the Sardine Phase and 1999-2004 in the Flying Fish Phase. The 1% down stage, reached at 100.8 +/- 8.8 d, is a stage of plumage development at which growth is essentially complete^1^ (Apanius et al. 2008) and only 1% of nestling down has not yet been replaced by pennaceous juvenile plumage. Adoptions and all cases involved in experimental brood size manipulations were excluded from the dataset. We included offspring raised by one or more banded parents and controlled the non-independence of repeated measurements by running a linear mixed model with pair identity fit as a random effect; unbanded individuals paired with a banded mate were assumed to be the same individual when assigning pair identity across years. Breeding season was included as an additional random effect. Fixed effects included Fish Phase (a two-level factor) and sea surface temperature anomalies (SSTA) averaged over two periods (December-February and April-June; see below). Statistical significance of the Fish Phase predictor was evaluated with a likelihood ratio test. Excluding 16 records with anomalously long periods to reach 1% down (>135 d) did not alter the Fish Phase coefficient (data not shown). Estimated age at 1% down by Fish Phase was also robust to using a much smaller dataset of 1,159 offspring where both parents were of known identity, modelled as above, but including separate random effects for mother and father identity and additional linear and quadratic terms for mother and father age (estimated as year of banding + 4 for parents banded as adults; FP (Sardine) β_N = 4075_ = -8.31[-13.4, -3.1], FP (Sardine) β_N = 1159_ = -8.31[-13.9, -2.89]).
