# Supplementary figures and images for: Decadal-scale variation in diet forecasts persistently poor breeding under ocean warming in a tropical seabird
Source: PLoS One. 2017 Aug 23;12(8):e0182545. doi: 10.1371/journal.pone.0182545 (PMC5568137; doi:10.1371/journal.pone.0182545)

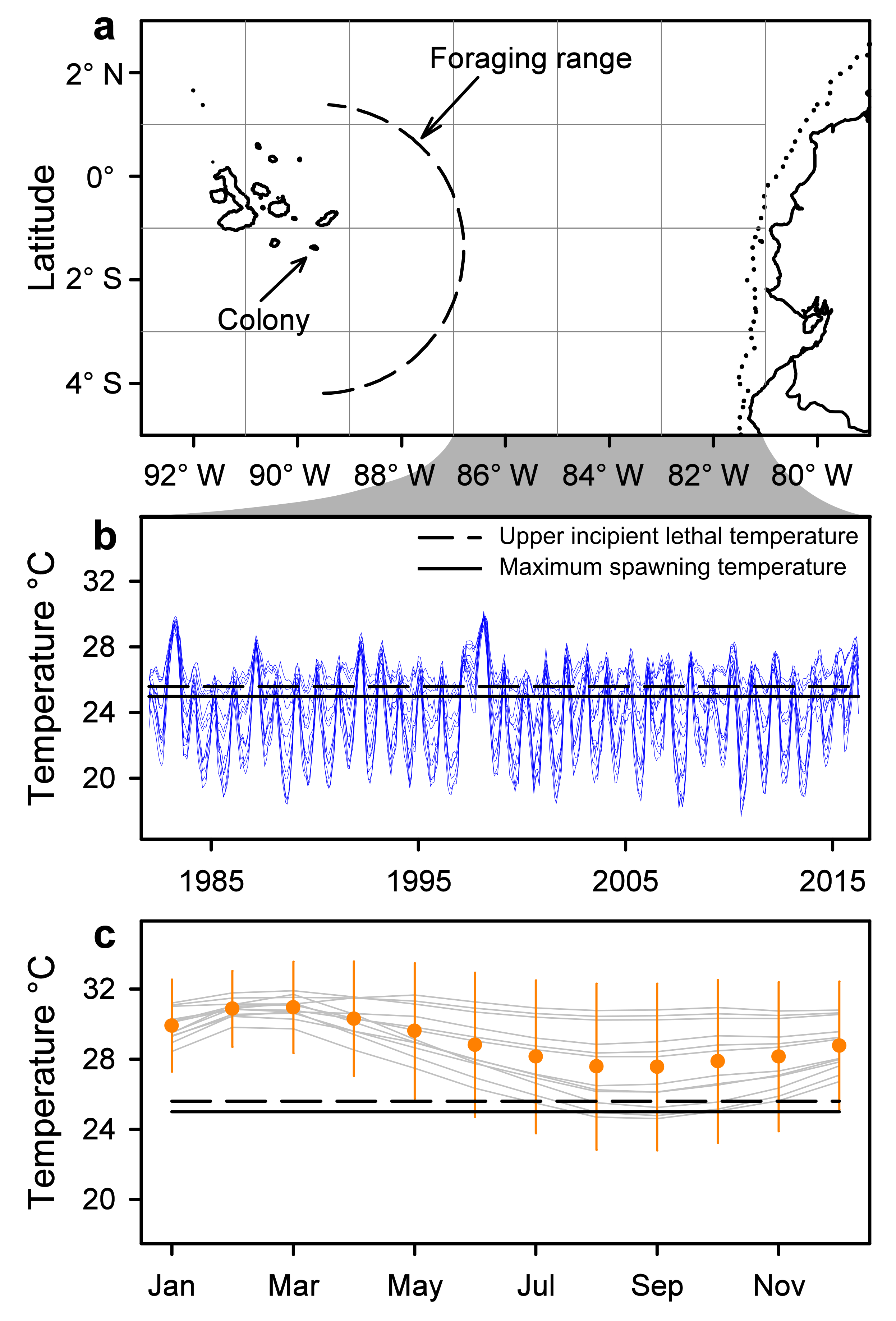

Supplement: S1 Fig — (a) Foraging envelope [48] (semicircle) of breeding Nazca boobies from Isla Española, Galápagos. Most present-day trips are within the area of the semicircle. Dotted line to the east shows the continental shelf break (1000 m isobath), roughly indicating the distribution of continental Pacific sardines, a probable source population for Galápagos. (b) Recent temporal variation (blue lines) in SST in each of 12 2° x 2° blocks east of the present-day foraging range of Nazca boobies. Solid horizontal line shows upper limit of spawning SST range of Pacific sardines (25°C; [45–47]). Dashed horizontal line shows upper incipient lethal limit for warm-acclimated temperate-origin Pacific sardines (25.6°C; [44]). (c) Recent SST from (a) with 4.5°C warming expected within 100 years. Orange pts (± 2 S.D.) show expected temperature averaged across all 12 blocks and across all years (1982–2016) by month. Gray lines show temperatures averaged across all years, by month, for each individual block. Monthly SST values for each 2° x 2° block were downloaded on 12 May 2016 from http://iridl.ldeo.columbia.edu/SOURCES/.NOAA/.NCDC/.ERSST/.version3b/.sst/. (TIF) [file pone.0182545.s002.tif]

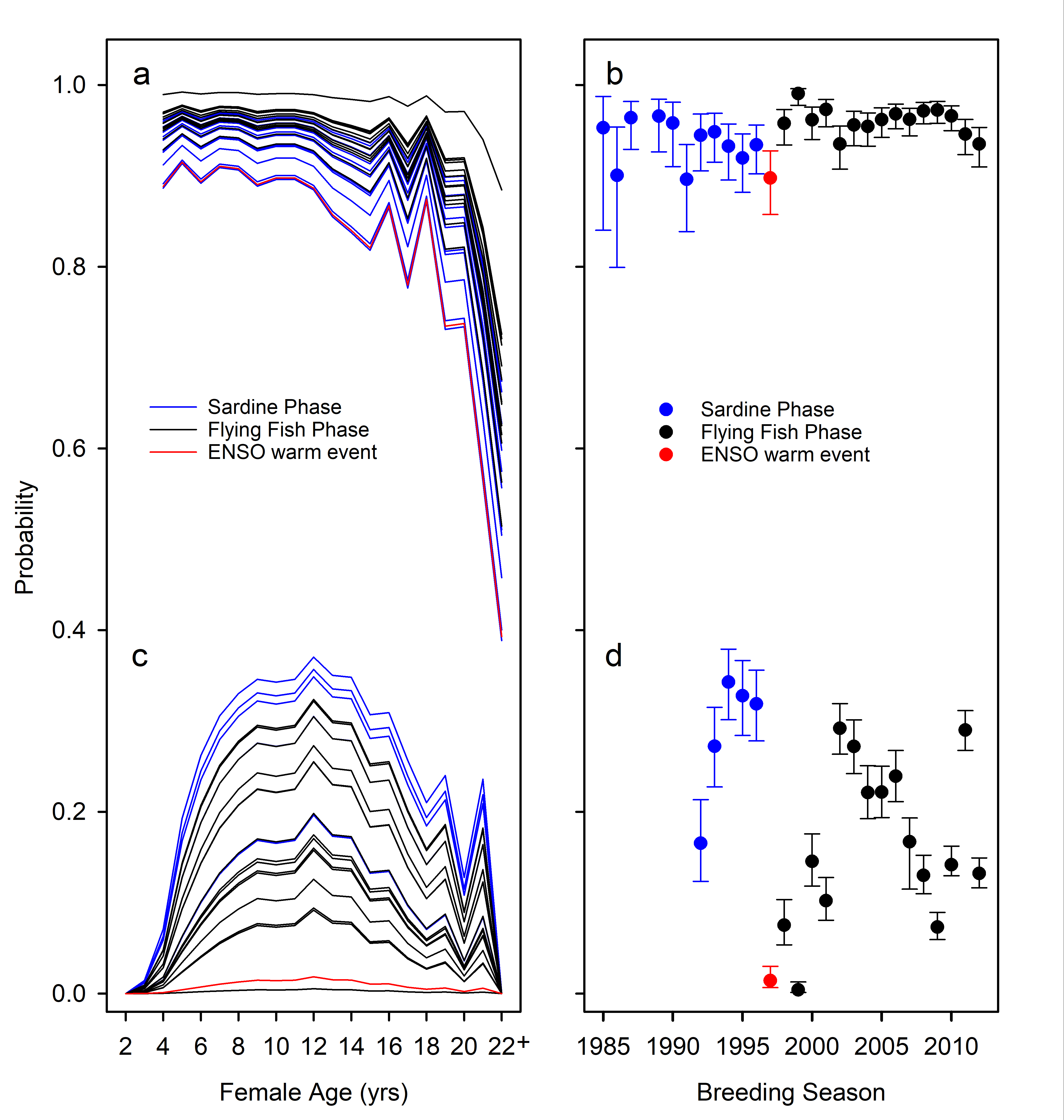

Supplement: S2 Fig — (a) Age-specific annual survival probabilities of adult females (N = 3,332 individuals) for each of the years 1985–2012 estimated with a mark-recapture model controlling encounter probabilities <1; year and age fit additively as multi-level factors (ages 4–21, 22+). (b) Temporal sequence of annual survival probabilities holding age constant at 10 yrs; brackets show 95% CIs. (c) Age-specific Annual Breeding Success (N = 14,640 breeding records) for 1992–2006, 2008–2012 from a binomial GLMM (logit link); year and age fit additively as multi-level factors (ages 2–21, 22+). In 2007, value was estimated using annual survival (see S1 Methods). (d) Temporal sequence of Annual Breeding Success holding age constant at 10 yrs; brackets show 95% CIs. (TIF) [file pone.0182545.s003.tif]

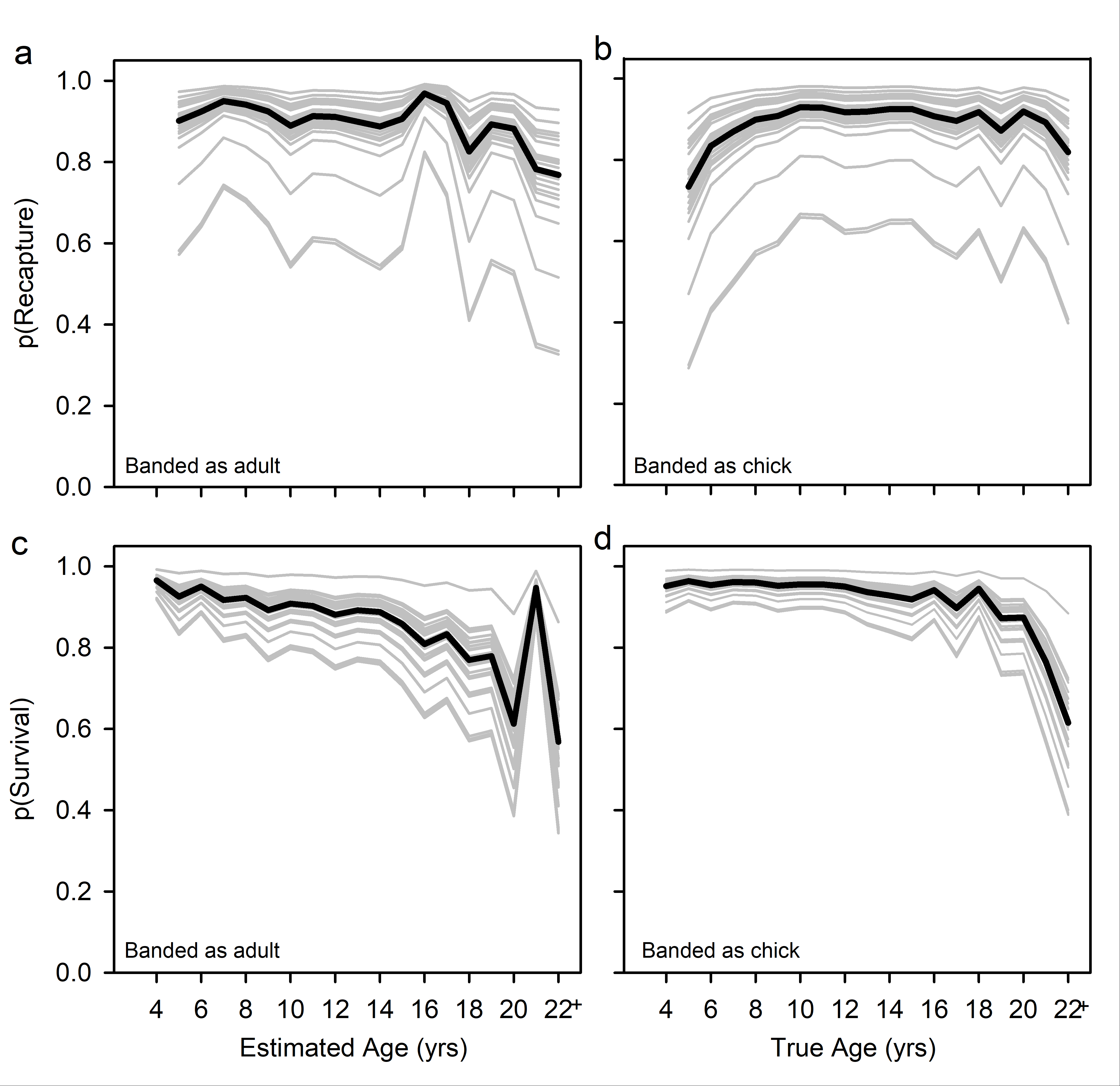

Supplement: S3 Fig — Estimated age assigned as 4 in the year of banding for individuals banded as adults. Survival and recapture probabilities from the top mark-recapture model allowing both probabilities to vary by year and by banding class (as adult or as young of the year) interacting with age (multi-level factor, levels 4–21, 22+). Values for individual years 1985–2012 are in grey, with the median age-specific trajectory marked by a thick black line. (TIF) [file pone.0182545.s004.tif]

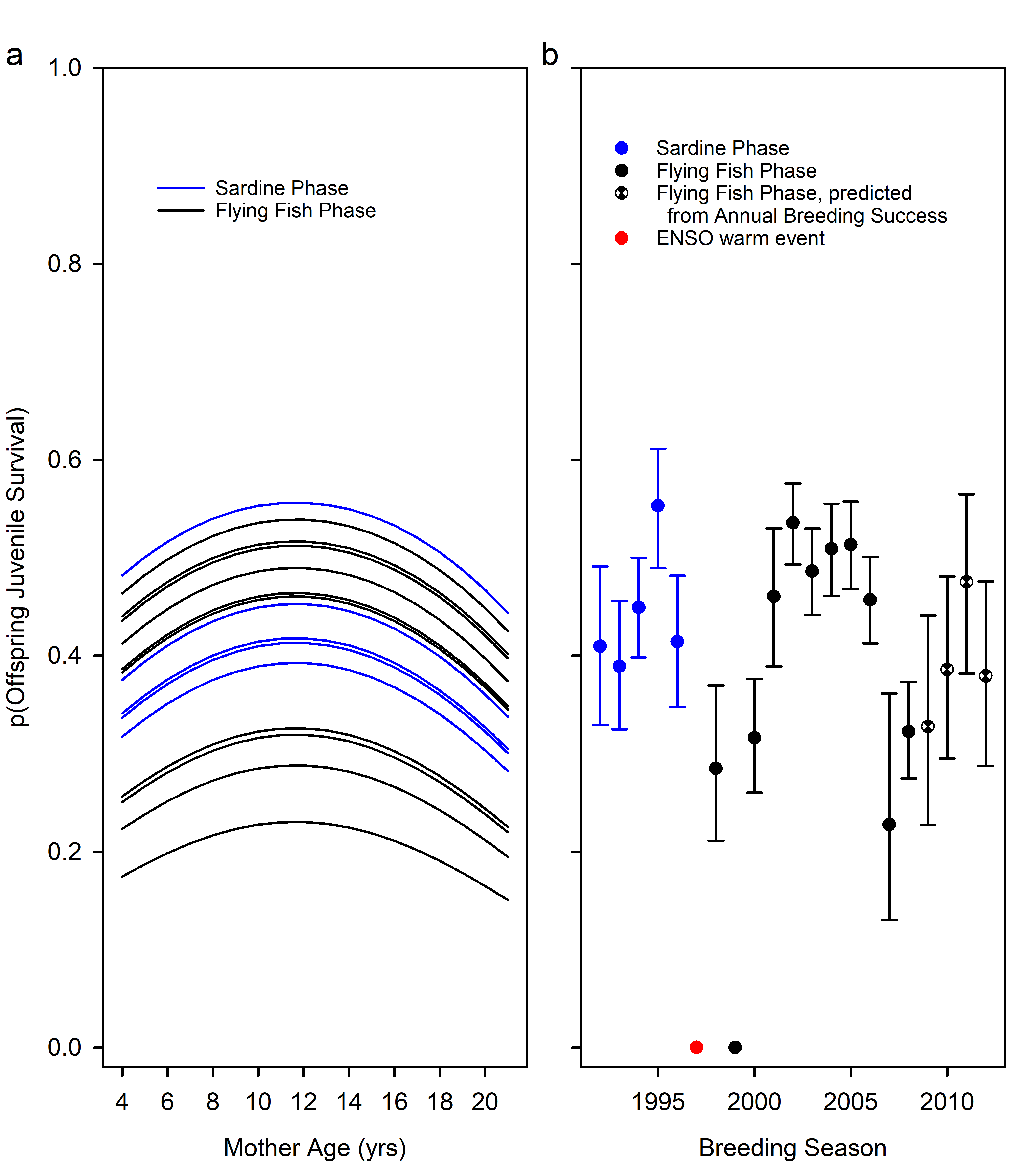

Supplement: S4 Fig — (a) Curves are predictions for each year from a GLMM (binomial errors, logit link) modeling offspring survival from independence to recruitment by mother’s age (continuous; linear and quadratic predictors) and year (factor, 1992–1996, 1998, 2000–2008), N = 3,813. (b) Temporal sequence of annual values holding a mother’s age constant at 10 yrs. Checkered points (2009–2012) mark years too recent for cohort members to have fully recruited; for these years, annual rate of juvenile survival was predicted from its positive relationship with Annual Breeding Success. Brackets show 95% CIs. Offspring sex was unknown for some cohorts, so the sexes were combined for modeling and all values were adjusted for the 33% lower survivorship of female offspring compared to male [51]. Few offspring reached independence in 1997 (N = 18) and 1999 (N = 10) and none survived the juvenile period, preventing inclusion of these years in the GLMM. (TIF) [file pone.0182545.s005.tif]

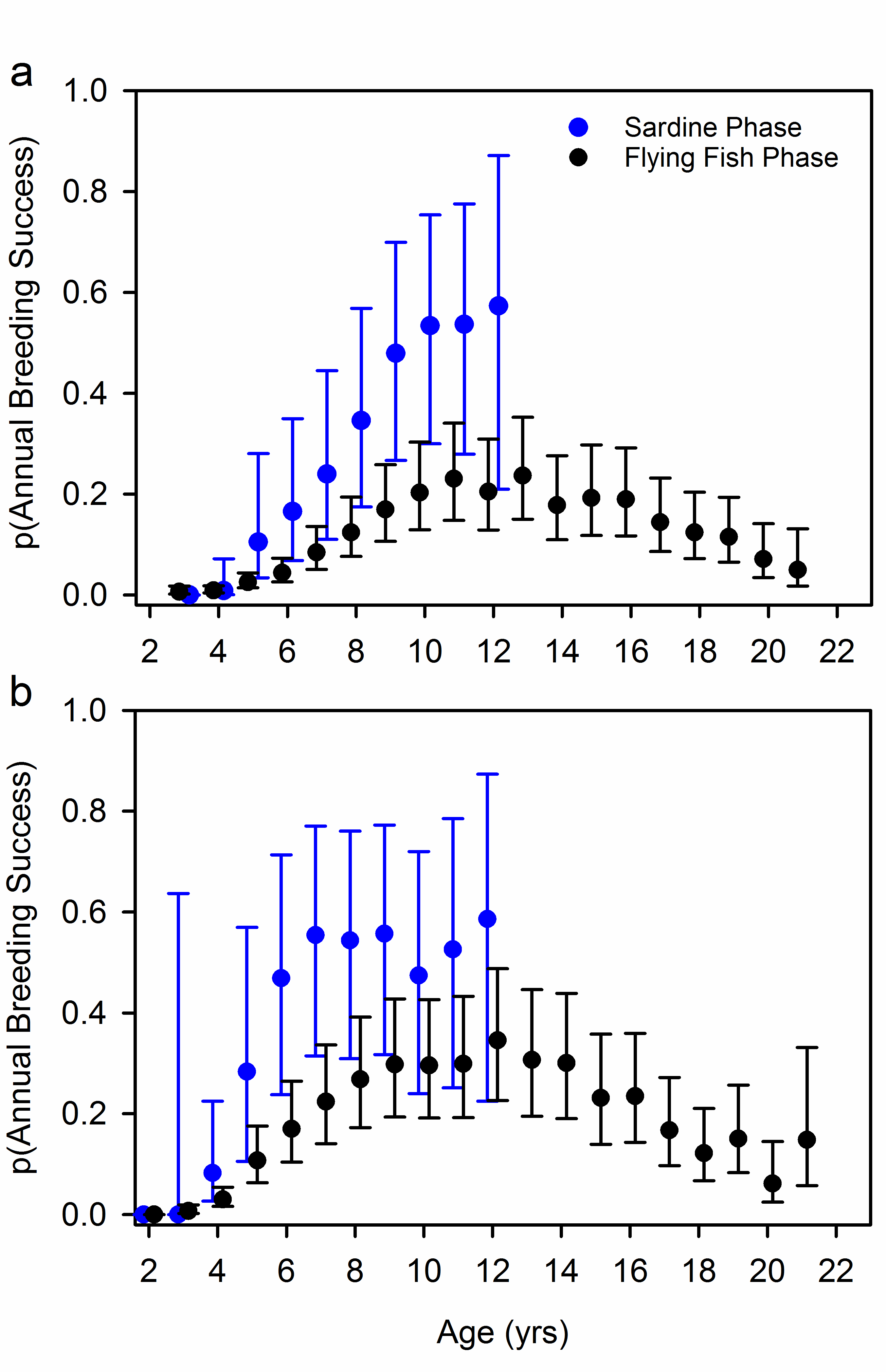

Supplement: S5 Fig — Breeding records of (a) males (N = 19,635 breeding records) and (b) females (N = 14,302 breeding records) during five seasons of Sardine Phase (1992–1996) and 14 seasons of Flying Fish Phase (1998–2012). Predicted mean Annual Breeding Success (95% CIs) by age (fit as a multi-level factor), Fish Phase, and their interaction, with year and identity random effects from sex-specific GLMMs (binomial errors, logit link). Sardine Phase age classes truncated at 12 because the diet switch occurred before old adults were produced by earlier banding of young of the year. (TIF) [file pone.0182545.s006.tif]
