# Supplementary material for: Decadal-scale variation in diet forecasts persistently poor breeding under ocean warming in a tropical seabird
Source: PLoS One. 2017 Aug 23;12(8):e0182545. doi: 10.1371/journal.pone.0182545 (PMC5568137; doi:10.1371/journal.pone.0182545)
Supplement: S1 Table — Models within ΔAICc of 2 are considered to be highly supported (in bold) unless they are more complex, nested versions of the top model. Such a model is penalized only 2 AIC units for each additional term and appears to be well-supported despite little variance explained by the additional fixed effect(s). Models ΔAICc ≥ 7 from the top model for each response variable are not presented. FP: a dichotomous factor for Fish Phase; SSTAAMJ and SSTADJF: local sea surface temperature anomalies averaged across Apr-Jun and Dec-Feb, respectively; El Niño: a dichotomous factor marking the 1997–98 extreme ENSO warm event. Main effects (Age + FP) plus the interaction between Age and FP are written as “Age x FP”. All models included female identity and year as random effects. The number of parameters (k), small sample size-corrected AIC value (AICc), AICc difference from the top model (ΔAICc), and Akaike weights (ωi) are reported. (DOCX) [file pone.0182545.s007.docx]

**S1 Table. Model selection using AICc to rank GLMMs (binomial errors, logit link) explaining variation in Annual Breeding Success and sequential reproductive stages in young and middle-aged (< 11 yrs) female Nazca boobies.** Models within ΔAICc of 2 are considered to be highly supported (in bold) unless they are more complex, nested versions of the top model. Such a model is penalized only 2 AIC units for each additional term and appears to be well-supported despite little variance explained by the additional fixed effect(s). Models ΔAICc > 7 from the top model for each response variable are not presented. FP: a dichotomous factor for Fish Phase; SSTA_AMJ_ and SSTA_DJF_: local sea surface temperature anomalies averaged across Apr-Jun and Dec-Feb, respectively; *El Niño*: a dichotomous factor marking the 1997-98 extreme ENSO warm event. Main effects (Age + FP) plus the interaction between Age and FP are written as “Age x FP”. All models included female identity and year as random effects. The number of parameters (*k*), small sample size-corrected AIC value (AICc), AICc difference from the top model (ΔAICc), and Akaike weights (*ω_i_*) are reported.

| **Model** | ***k*** | **AICc** | **ΔAICc** | **ω*i*** |
| --- | --- | --- | --- | --- |
| Annual Breeding Success (all years) |  |  |  |  |
| **SSTA_DJF_ + El Niño + FP + Age + Age^2^** | **8** | **11,119.7** | **0** | **0.33** |
| SSTA_DJF_ + El Niño + Age x FP + Age^2^ | 9 | 11,121.4 | 1.65 | 0.15 |
| SSTA_AMJ_ + SSTA_DJF_ + El Niño + FP + Age + Age^2^ | 9 | 11,121.7 | 2.00 | 0.12 |
| El Niño + FP + Age + Age^2^ | 7 | 11,121.9 | 2.17 | 0.11 |
| SSTA_DJF_ + El Niño + Age x FP + Age^2^ | 7 | 11,123.3 | 3.53 | 0.06 |
| SSTA_AMJ_ + SSTA_DJF_ + El Niño + Age x FP + Age^2^ | 10 | 11,123.4 | 3.65 | 0.05 |
| El Niño + Age x FP + Age^2^ | 8 | 11,123.6 | 3.89 | 0.05 |
| SSTA_AMJ_ + El Niño + FP + Age + Age^2^ | 8 | 11,123.8 | 4.09 | 0.04 |
| El Niño + Age + Age^2^ | 6 | 11,125.0 | 5.26 | 0.02 |
| SSTA_AMJ_ + SSTA_DJF_ + El Niño + Age + Age^2^ | 8 | 11,125.0 | 5.26 | 0.02 |
| SSTA_AMJ_ + El Niño + Age x FP + Age^2^ | 9 | 11,125.6 | 5.82 | 0.02 |
| Annual Breeding Success (12 yrs) |  |  |  |  |
| **SSTA_DJF_ + El Niño + FP + Age + Age^2^** | **8** | **3,578.9** | **0** | **0.40** |
| SSTA_DJF_ + El Niño + Age x FP + Age^2^ | 9 | 3,579.6 | 0.75 | 0.27 |
| SSTA_AMJ_ + SSTA_DJF_ + El Niño + FP + Age + Age^2^ | 9 | 3,580.8 | 1.99 | 0.15 |
| SSTA_AMJ_ + SSTA_DJF_ + El Niño + Age x FP + Age^2^ | 10 | 3,581.6 | 2.71 | 0.10 |
| SSTA_DJF_ + El Niño + Age + Age^2^ | 7 | 3,584.1 | 5.22 | 0.03 |
| p(lay \| alive) (12 seasons) |  |  |  |  |
| **SSTA_DJF_ + El Niño + Age + Age^2^** | **7** | **3,094.2** | **0** | **0.30** |
| **SSTA_DJF_ + Age + Age^2^** | **6** | **3,095.3** | **1.09** | **0.17** |
| SSTA_DJF_ + El Niño + FP + Age + Age^2^ | 8 | 3,095.7 | 1.42 | 0.15 |
| Age + Age^2^ | 5 | 3,096.5 | 2.22 | 0.10 |
| SSTA_DJF_ + FP + Age + Age^2^ | 7 | 3,097.2 | 2.96 | 0.07 |
| SSTA_DJF_ + El Niño + Age x FP + Age^2^ | 9 | 3,097.6 | 3.32 | 0.06 |
| FP + Age + Age^2^ | 6 | 3,097.9 | 3.67 | 0.05 |
| El Niño + Age + Age^2^ | 6 | 3,098.2 | 3.91 | 0.04 |
| SSTA_DJF_ + Age x FP + Age^2^ | 8 | 3,099.1 | 4.88 | 0.03 |
| El Niño + FP + Age + Age^2^ | 7 | 3,099.8 | 5.55 | 0.02 |
| Age x FP + Age^2^ | 7 | 3,099.8 | 5.56 | 0.02 |
| p(hatch \| lay) (12 seasons) |  |  |  |  |
| **SSTA_DJF_ + El Niño + FP + Age + Age^2^** | **8** | **3,127.8** | **0** | **0.66** |
| SSTA_DJF_ + El Niño + Age x FP + Age^2^ | 9 | 3,129.7 | 1.90 | 0.26 |
| p(independent offspring \| hatch) (12 yrs) |  |  |  |  |
| **SSTA_DJF_ + El Niño + FP + Age + Age^2^** | **8** | **1,927.3** | **0** | **0.38** |
| SSTA_AMJ_ + SSTA_DJF_ + El Niño + FP + Age + Age^2^ | 9 | 1,928.7 | 1.39 | 0.19 |
| SSTA_DJF_ + El Niño + Age x FP + Age^2^ | 9 | 1,929.1 | 1.73 | 0.16 |
| SSTA_AMJ_ + SSTA_DJF_ + El Niño + Age x FP + Age^2^ | 10 | 1,930.3 | 2.99 | 0.08 |
| El Niño + FP + Age + Age^2^ | 7 | 1,930.9 | 3.61 | 0.06 |
| SSTA_AMJ_ + El Niño + FP + Age + Age^2^ | 8 | 1,931.5 | 4.16 | 0.05 |
| El Niño + Age x FP + Age^2^ | 8 | 1,932.2 | 4.86 | 0.03 |
| SSTA_AMJ_ + El Niño + Age x FP + Age^2^ | 9 | 1,932.5 | 5.15 | 0.03 |
