# Supplementary material for: Decadal-scale variation in diet forecasts persistently poor breeding under ocean warming in a tropical seabird
Source: PLoS One. 2017 Aug 23;12(8):e0182545. doi: 10.1371/journal.pone.0182545 (PMC5568137; doi:10.1371/journal.pone.0182545)
Supplement: S2 Table — (DOCX) [file pone.0182545.s008.docx]

**S2 Table. Results as in S1 Table, but for young and middle-aged male Nazca boobies.**

| **Model** | ***k*** | **AICc** | **ΔAICc** | **ω*_i_*** |
| --- | --- | --- | --- | --- |
| Annual Breeding Success (19 yrs) |  |  |  |  |
| **SSTA_DJF_ + El Niño + FP + Age + Age^2^** | **8** | **11,745.9** | **0** | **0.25** |
| SSTA_DJF_ + El Niño + Age x FP + Age^2^ | 9 | 11,746.0 | 0.10 | 0.23 |
| **El Niño + FP + Age + Age^2^** | **7** | **11,747.3** | **1.41** | **0.12** |
| SSTA_AMJ_ + SSTA_DJF_ + El Niño + FP + Age + Age^2^ | 9 | 11,747.7 | 1.78 | 0.10 |
| El Niño + FP + Age x FP + Age^2^ | 8 | 11,747.8 | 1.91 | 0.10 |
| SSTA_AMJ_ + SSTA_DJF_ + El Niño + Age x FP + Age^2^ | 10 | 11,747.8 | 1.93 | 0.09 |
| SSTA_AMJ_ + El Niño + FP + Age + Age^2^ | 8 | 11,748.8 | 2.96 | 0.06 |
| SSTA_AMJ_ + El Niño + Age x FP + Age^2^ | 9 | 11,749.4 | 3.50 | 0.04 |
| Annual Breeding Success (12 yrs) |  |  |  |  |
| **SSTA_AMJ_ + SSTA_DJF_ + El Niño + FP + Age + Age^2^** | **9** | **3,964.2** | **0** | **0.50** |
| SSTA_AMJ_ + SSTA_DJF_ + El Niño + Age x FP + Age^2^ | 10 | 3,965.5 | 1.32 | 0.26 |
| SSTA_DJF_ + El Niño + FP + Age + Age^2^ | 8 | 3,966.3 | 2.10 | 0.17 |
| SSTA_DJF_ + El Niño + Age x FP + Age^2^ | 9 | 3,968.3 | 4.07 | 0.07 |
| p(lay \| alive) (12 seasons) |  |  |  |  |
| **SSTA_DJF_ + El Niño + FP + Age + Age^2^** | **8** | **5,268.9** | **0** | **0.54** |
| SSTA_DJF_ + El Niño + Age x FP + Age^2^ | 9 | 5,270.4 | 1.54 | 0.25 |
| FP + Age + Age^2^ | 6 | 5,273.4 | 4.50 | 0.06 |
| El Niño + FP + Age + Age^2^ | 7 | 5,274.2 | 5.39 | 0.04 |
| SSTA_DJF_ + FP + Age + Age^2^ | 7 | 5,275.1 | 6.27 | 0.02 |
| Age x FP + Age^2^ | 7 | 5,275.3 | 6.45 | 0.02 |
| SSTA_DJF_ + El Niño + Age + Age^2^ | 7 | 5,275.6 | 6.73 | 0.02 |
| p(hatch \| lay) (12 seasons) |  |  |  |  |
| **SSTA_DJF_ + El Niño + FP + Age + Age^2^** | **8** | **2,805.6** | **0** | **0.42** |
| SSTA_DJF_ + El Niño + Age x FP + Age^2^ | 9 | 2,807.4 | 1.79 | 0.17 |
| SSTA_DJF_ + El Niño + FP + Age | 7 | 2,807.9 | 2.36 | 0.13 |
| El Niño + FP + Age + Age^2^ | 7 | 2,808.3 | 2.67 | 0.11 |
| SSTA_DJF_ + El Niño + Age x FP | 8 | 2,809.3 | 3.68 | 0.07 |
| El Niño + Age x FP + Age^2^ | 8 | 2,810.3 | 4.67 | 0.04 |
| El Niño + FP + Age | 6 | 2,811.3 | 5.70 | 0.02 |
| p(independent offspring \| hatch) (12 yrs) |  |  |  |  |
| **SSTA_AMJ_ + SSTA_DJF_ + El Niño + FP + Age** | **8** | **1,665.7** | **0** | **0.36** |
| SSTA_AMJ_ + SSTA_DJF_ + El Niño + Age x FP | 9 | 1,667.3 | 1.57 | 0.16 |
| SSTA_AMJ_ + SSTA_DJF_ + El Niño + FP + Age + Age^2^ | 9 | 1,667.4 | 1.68 | 0.16 |
| SSTA_AMJ_ + SSTA_DJF_ + El Niño + Age x FP + Age^2^ | 10 | 1,669.1 | 3.41 | 0.07 |
| SSTA_DJF_ + El Niño + FP + Age | 7 | 1,669.3 | 3.58 | 0.06 |
| SSTA_AMJ_ + El Niño + FP + Age | 7 | 1,669.5 | 3.80 | 0.05 |
| SSTA_DJF_ + El Niño + FP + Age + Age^2^ | 8 | 1,670.4 | 4.67 | 0.04 |
| SSTA_DJF_ + El Niño + Age x FP | 8 | 1,670.8 | 5.08 | 0.03 |
| SSTA_AMJ_ + El Niño + FP + Age + Age^2^ | 8 | 1,671.3 | 5.57 | 0.02 |
| SSTA_AMJ_ + El Niño + Age x FP | 8 | 1,671.4 | 5.68 | 0.02 |
| SSTA_DJF_ + El Niño + Age x FP + Age^2^ | 9 | 1,672.2 | 6.43 | 0.01 |
