# Supplementary material for: Decadal-scale variation in diet forecasts persistently poor breeding under ocean warming in a tropical seabird
Source: PLoS One. 2017 Aug 23;12(8):e0182545. doi: 10.1371/journal.pone.0182545 (PMC5568137; doi:10.1371/journal.pone.0182545)
Supplement: S3 Table — Models within ΔAICc of 2 are considered to be highly supported (in bold) unless they are more complex, nested versions of the top model. Such a model is penalized only 2 AIC units for each additional term and appears to be well-supported despite little variance explained by the additional fixed effect(s). Models ΔAICc ≥ 7 from the top model for each response variable are not presented. YBD: centered, continuous, years before death; FP: a dichotomous factor for Fish Phase; SSTAAMJ and SSTADJF: local sea surface temperature anomalies averaged across Apr-Jun and Dec-Feb, respectively. Main effects (YBD + FP) plus the interaction between YBD and FP are written as “YBD x FP”. All models included female identity and year as random effects. The number of parameters (k), small sample size-corrected AIC value (AICc), AICc difference from the top model (ΔAICc), and Akaike weights (ωi) are reported. (DOCX) [file pone.0182545.s009.docx]

**S3 Table. Model selection using AICc to rank GLMMs (binomial errors, logit link) explaining variation in Annual Breeding Success and sequential reproductive stages in presumed old female Nazca boobies.** Models within ΔAICc of 2 are considered to be highly supported (in bold) unless they are more complex, nested versions of the top model. Such a model is penalized only 2 AIC units for each additional term and appears to be well-supported despite little variance explained by the additional fixed effect(s). Models ΔAICc > 7 from the top model for each response variable are not presented. YBD: centered, continuous, years before death; FP: a dichotomous factor for Fish Phase; SSTA_AMJ_ and SSTA_DJF_: local sea surface temperature anomalies averaged across Apr-Jun and Dec-Feb, respectively. Main effects (YBD + FP) plus the interaction between YBD and FP are written as “YBD x FP”. All models included female identity and year as random effects. The number of parameters (*k*), small sample size-corrected AIC value (AICc), AICc difference from the top model (ΔAICc), and Akaike weights (*ω_i_*) are reported.

| **Model** | ***k*** | **AICc** | | **ΔAICc** | | **ω*_i_*** | |  |
| --- | --- | --- | --- | --- | --- | --- | --- | --- |
| Annual Breeding Success (18 yrs) |  |  | |  | |  | |  |
| **SSTA_DJF_ + FP + YBD** | **6** | **2,484.1** | | **0** | | **0.35** | |  |
| **FP + YBD** | **5** | **2,485.7** | | **1.55** | | **0.16** | |  |
| SSTA_AMJ_ + SSTA_DJF_ + FP + YBD | 7 | 2,486.1 | | 1.94 | | 0.13 | |  |
| SSTA_DJF_ + FP x YBD | 7 | 2,486.1 | | 1.94 | | 0.13 | |  |
| FP x YBD | 6 | 2,487.5 | | 3.41 | | 0.06 | |  |
| SSTA_AMJ_ + FP + YBD | 6 | 2,487.7 | | 3.56 | | 0.06 | |  |
| SSTA_AMJ_ + SSTA_DJF_ + FP x YBD | 8 | 2,488.0 | | 3.88 | | 0.05 | |  |
| SSTA_AMJ_ + FP x YBD | 7 | 2,489.5 | | 5.42 | | 0.02 | |  |
| SSTA_DJF_ + YBD | 5 | 2,490.4 | | 6.32 | | 0.02 | |  |
| Annual Breeding Success (11 yrs) |  |  | |  | |  | |  |
| **SSTA_DJF_ + FP + YBD** | **6** | **1,298.5** | | **0** | | **0.47** | |  |
| SSTA_DJF_ + FP x YBD | 7 | 1,300.3 | | 1.75 | | 0.20 | |  |
| SSTA_AMJ_ + SSTA_DJF_ + FP + YBD | 7 | 1,300.5 | | 2.01 | | 0.17 | |  |
| SSTA_AMJ_ + SSTA_DJF_ + FP x YBD | 8 | 1,302.3 | | 3.75 | | 0.07 | |  |
| SSTA_DJF_ + YBD | 5 | 1,303.6 | | 5.08 | | 0.04 | |  |
| SSTA_AMJ_ + SSTA_DJF_ + YBD | 6 | 1,305.3 | | 6.79 | | 0.02 | |  |
| FP + YBD | 5 | 1,305.6 | | 7.07 | | 0.01 | |  |
| p(lay \| alive) (11 yrs) |  |  | |  | |  | |  |
| **SSTA_DJF_ + FP x YBD** | **7** | **1,000.4** | | **0** | | **0.36** | |  |
| **SSTA_DJF_ + YBD** | **5** | **1,001.0** | | **0.55** | | **0.28** | |  |
| SSTA_DJF_ + FP + YBD | 6 | 1,001.2 | | 0.76 | | 0.25 | |  |
| YBD | 4 | 1,004.2 | | 3.75 | | 0.06 | |  |
| FP + YBD | 5 | 1,005.1 | | 4.7 | | 0.04 | |  |
| FP x YBD | 6 | 1,006.3 | | 5.87 | | 0.02 | |  |
| p(hatch \| lay) (11 yrs) |  |  | |  | |  | |  |
| **SSTA_DJF_ + FP + YBD** | **6** | **1,155.6** | | **0** | | **0.65** | |  |
| SSTA_DJF_ + FP x YBD | 7 | 1,157.3 | | 1.69 | | 0.28 | |  |
| SSTA_DJF_ + YBD | 5 | 1,161.9 | | 6.30 | | 0.03 | |  |
| FP + YBD | 5 | 1,162.4 | | 6.79 | | 0.02 | |  |
| p(independent offspring \| hatch) (11 yrs) | |  |  | |  | |  | |
| **SSTA_DJF_ + FP** | **5** | **719.9** | | **0** | | **0.34** | |  |
| SSTA_DJF_ + FP + YBD | 6 | 721.1 | | 1.16 | | 0.19 | |  |
| SSTA_AMJ_ + SSTA_DJF_ + FP | 6 | 722.0 | | 2.02 | | 0.12 | |  |
| SSTA_DJF_ + FP x YBD | 7 | 722.9 | | 2.92 | | 0.08 | |  |
| SSTA_AMJ_ + SSTA_DJF_ + FP + YBD | 7 | 723.1 | | 3.19 | | 0.07 | |  |
| FP | 4 | 724.0 | | 4.03 | | 0.05 | |  |
| SSTA_AMJ_ + SSTA_DJF_ + FP x YBD | 8 | 724.9 | | 4.95 | | 0.03 | |  |
| FP + YBD | 5 | 725.0 | | 5.07 | | 0.03 | |  |
| SSTA_DJF_ | 4 | 725.6 | | 5.66 | | 0.02 | |  |
| SSTA_AMJ_ + FP | 5 | 725.8 | | 5.82 | | 0.02 | |  |
| SSTA_DJF_ + YBD | 5 | 726.6 | | 6.67 | | 0.01 | |  |
| SSTA_AMJ_ + FP + YBD | 6 | 726.8 | | 6.87 | | 0.01 | |  |
| Intercept | 3 | 726.9 | | 6.95 | | 0.01 | |  |
