# Supplementary material for: Decadal-scale variation in diet forecasts persistently poor breeding under ocean warming in a tropical seabird
Source: PLoS One. 2017 Aug 23;12(8):e0182545. doi: 10.1371/journal.pone.0182545 (PMC5568137; doi:10.1371/journal.pone.0182545)
Supplement: S4 Table — (DOCX) [file pone.0182545.s010.docx]

**S4 Table. Results as in S3 Table, but for presumed old male Nazca boobies.**

| **Model** | ***k*** | **AICc** | **ΔAICc** | **ω*_i_*** |
| --- | --- | --- | --- | --- |
| Annual Breeding Success (18 yrs) |  |  |  |  |
| **SSTA_DJF_ + FP + YBD** | **6** | **2,746.8** | **0** | **0.26** |
| **FP + YBD** | **5** | **2,747.1** | **0.29** | **0.23** |
| SSTA_AMJ_ + SSTA_DJF_ + FP + YBD | 7 | 2,748.4 | 1.56 | 0.12 |
| SSTA_AMJ_ + FP + YBD | 6 | 2,748.4 | 1.57 | 0.12 |
| SSTA_DJF_ + FP x YBD | 7 | 2,748.8 | 2.01 | 0.10 |
| FP x YBD | 6 | 2,749.1 | 2.29 | 0.08 |
| SSTA_AMJ_ + FP x YBD | 7 | 2,750.4 | 3.57 | 0.04 |
| SSTA_AMJ_ + SSTA_DJF_ + FP x YBD | 8 | 2,750.4 | 3.57 | 0.04 |
| Annual Breeding Success (11 yrs) |  |  |  |  |
| **SSTA_DJF_ + FP + YBD** | **6** | **1,404.4** | **0** | **0.40** |
| SSTA_AMJ_ + SSTA_DJF_ + FP + YBD | 7 | 1,405.5 | 1.12 | 0.23 |
| SSTA_DJF_ + FP x YBD | 7 | 1,405.6 | 1.12 | 0.23 |
| SSTA_AMJ_ + SSTA_DJF_ + FP x YBD | 8 | 1,406.8 | 2.34 | 0.13 |
| p(lay \| alive) (11 yrs) |  |  |  |  |
| **SSTA_DJF_ + FP + YBD** | **6** | **1,817.4** | **0** | **0.47** |
| SSTA_DJF_ + FP x YBD | 7 | 1,818.5 | 1.05 | 0.28 |
| FP + YBD | 5 | 1,820.3 | 2.90 | 0.11 |
| SSTA_DJF_ + YBD | 5 | 1,821.5 | 4.10 | 0.06 |
| FP x YBD | 6 | 1,822.0 | 4.56 | 0.05 |
| YBD | 4 | 1,822.7 | 5.25 | 0.03 |
| p(hatch \| lay) (11 yrs) |  |  |  |  |
| **SSTA_DJF_ + FP** | **5** | **916.3** | **0** | **0.53** |
| SSTA_DJF_ + FP + YBD | 6 | 917.6 | 1.30 | 0.28 |
| SSTA_DJF_ + FP x YBD | 7 | 919.4 | 3.10 | 0.11 |
| FP | 4 | 922.3 | 6.02 | 0.03 |
| SSTA_DJF_ | 4 | 923.3 | 6.93 | 0.02 |
| p(independent offspring \| hatch) (11 yrs) |  |  |  |  |
| **SSTA_DJF_ + FP + YBD** | **6** | **623.2** | **0** | **0.48** |
| SSTA_DJF_ + FP x YBD | 7 | 625.0 | 1.80 | 0.20 |
| SSTA_AMJ_ + SSTA_DJF_ + FP + YBD | 7 | 625.2 | 1.97 | 0.18 |
| SSTA_AMJ_ + SSTA_DJF_ + FP x YBD | 8 | 627.0 | 3.80 | 0.07 |
| FP + YBD | 5 | 629.0 | 5.77 | 0.03 |
