# Supplementary material for: Decadal-scale variation in diet forecasts persistently poor breeding under ocean warming in a tropical seabird
Source: PLoS One. 2017 Aug 23;12(8):e0182545. doi: 10.1371/journal.pone.0182545 (PMC5568137; doi:10.1371/journal.pone.0182545)
Supplement: S5 Table — Predictors include Year: a multi-level factor; Age: a multi-level factor (4–21, 22+); G: group, either banded-as-adult or banded-as-chick; P: Period, a three-level factor grouping the Sardine Phase (1984–1996), the 1997 El Niño, and the Flying Fish Phase (1998–2013); an alternate version of P, P2, had four levels 1984–1991, 1992–1996, 1997, and 1998–2013, allowing a test for differences in survival between Sardine Phase years for which we have reproductive data and systematic diet sampling vs. earlier years. The number of parameters (k), small sample size-corrected AIC value (AICc), AICc difference from the top model (ΔAICc), and Akaike weights (ωi) are reported for the complete model set. Main effects (G + Age) plus the interaction between G and Age are written as “G x Age”. (DOCX) [file pone.0182545.s011.docx]

**S5 Table. Model selection results for a mark-recapture analysis of adult female survival (*Φ*) and recapture probabilities (*p*) using Program MARK.** Predictors include Year: a multi-level factor; Age: a multi-level factor (4-21, 22+); G: group, either banded-as-adult or banded-as-chick; P: Period, a three-level factor grouping the Sardine Phase (1984-1996), the 1997 *El Niño*, and the Flying Fish Phase (1998-2013); an alternate version of P, P2, had four levels 1984-1991, 1992-1996, 1997, and 1998-2013, allowing a test for differences in survival between Sardine Phase years for which we have reproductive data and systematic diet sampling *vs.* earlier years. The number of parameters (*k*), small sample size-corrected AIC value (AICc), AICc difference from the top model (ΔAICc), and Akaike weights (*ω_i_*) are reported for the complete model set. Main effects (G + Age) plus the interaction between G and Age are written as “G x Age”.

| **Model** |  | ***k*** | **AICc** | **ΔAICc** | ***ω*_i_** |
| --- | --- | --- | --- | --- | --- |
| *Φ*(Year + G x Age) | *p*(Year + G x Age) | 130 | 26,703.3 | 0.00 | 1.00 |
| *Φ*(P + G x Age) | *p*(Year + G x Age) | 104 | 26,760.7 | 57.41 | 0.00 |
| *Φ*(P2 + G x Age) | *p*(Year + G x Age) | 105 | 26,762.4 | 59.11 | 0.00 |
| *Φ*(G x Age) | *p*(Year + G x Age) | 102 | 26,949.8 | 246.54 | 0.00 |
| *Φ*(Year) | *p*(Year + G x Age) | 93 | 27,031.3 | 328.06 | 0.00 |
| *Φ*(P) | *p*(Year + G x Age) | 67 | 27,098.7 | 395.42 | 0.00 |
| *Φ*(P2) | *p*(Year + G x Age) | 68 | 27,100.2 | 396.90 | 0.00 |
| *Φ*(Year + G x Age) | *p*(Year) | 95 | 27,202.3 | 499.04 | 0.00 |
| *Φ*(Intercept) | *p*(Year + G x Age) | 65 | 27,217.3 | 514.08 | 0.00 |
| *Φ*(P + G x Age) | *p*(Year) | 69 | 27,247.7 | 544.43 | 0.00 |
| *Φ*(P2 + G x Age) | *p*(Year) | 70 | 27,249.6 | 546.37 | 0.00 |
| *Φ*(G x Age) | *p*(Year) | 67 | 27,338.1 | 634.88 | 0.00 |
| *Φ*(Year) | *p*(Year) | 58 | 27,624.7 | 921.46 | 0.00 |
| *Φ*(P) | *p*(Year) | 32 | 27,680.2 | 976.92 | 0.00 |
| *Φ*(P2) | *p*(Year) | 33 | 27,681.7 | 978.47 | 0.00 |
| *Φ*(Intercept) | *p*(Year) | 30 | 27,787.2 | 1,083.93 | 0.00 |
