# Supplementary material for: Decadal-scale variation in diet forecasts persistently poor breeding under ocean warming in a tropical seabird
Source: PLoS One. 2017 Aug 23;12(8):e0182545. doi: 10.1371/journal.pone.0182545 (PMC5568137; doi:10.1371/journal.pone.0182545)
Supplement: S6 Table — FP: a dichotomous factor for Fish Phase; SSTAAMJ and SSTADJF: local sea surface temperature anomalies averaged across Apr-Jun and Dec-Feb, respectively. The variance for the random effects associated with each model are presented along with the intraclass correlation coefficient (ICC; the proportion of total variance not accounted for by fixed factors) in brackets. (DOCX) [file pone.0182545.s012.docx]

**S6 Table. Coefficient estimates from an LMM evaluating Nazca booby offspring growth rates (measured as offspring age at the 1% remaining down stage) by Fish Phase.** FP: a dichotomous factor for Fish Phase; SSTA_AMJ_ and SSTA_DJF_: local sea surface temperature anomalies averaged across Apr-Jun and Dec-Feb, respectively. The variance for the random effects associated with each model are presented along with the intraclass correlation coefficient (ICC; the proportion of total variance not accounted for by fixed factors) in brackets.

| **Fixed effects** | **Estimate [95% CI]** | ***P*** |
| --- | --- | --- |
| Intercept | 106.06 [102.40, 109.77] | *** |
| FP (Sardine) | -8.31 [-13.38, -3.12] | * |
| SSTA_DJF_ | -5.78 [-10.38, -1.57] | * |
| SSTA_AMJ_ | -0.26 [-5.82, 5.19] |  |
|  |  |  |
| **Random effects** | **Variance [ICC]** |  |
| Pair ID | 11.73 [0.15] |  |
| Year | 20.43 [0.26] |  |
| Residual | 46.36 |  |
| *R^2^_m_^1^* | 0.19 |  |
| *R^2^_c_^1^* | 0.52 |  |
| N | 4,075 |  |

*P*: 0 ‘***’ 0.001 ‘**’ 0.01 ‘*’ 0.05 ‘.’ 0.1 ‘ ’ 1

*^1^*Marginal *R^2^* (*R^2^_m_*), and conditional *R^2^* (*R^2^_c_*) estimates were calculated following Nakagawa S, Schielzeth HA. A general and simple method for obtaining R^2^ from generalized linear mixed-effects models. Methods in Ecology and Evolution. 2013; 4: 133-142.
