# Supplementary material for: Decadal-scale variation in diet forecasts persistently poor breeding under ocean warming in a tropical seabird
Source: PLoS One. 2017 Aug 23;12(8):e0182545. doi: 10.1371/journal.pone.0182545 (PMC5568137; doi:10.1371/journal.pone.0182545)
Supplement: S7 Table — Age: centered, continuous; FP: a dichotomous factor for Fish Phase; SSTAAMJ and SSTADJF: local sea surface temperature anomalies averaged across Apr-Jun and Dec-Feb, respectively; El Niño: a dichotomous factor marking the 1997–98 extreme ENSO warm event. SSTAAMJ is not included as a predictor for traits expressed prior to April. The variance for the random effects associated with each model are presented along with the intraclass correlation coefficient (ICC; the proportion of total variance not accounted for by fixed factors) in brackets. (DOCX) [file pone.0182545.s013.docx]

**S7 Table. Coefficient estimates for the GLMM (binomial errors, logit link) best explaining variation (lowest AICc value; the “top model”) in Annual Breeding Success and sequential reproductive stages in young/middle-aged (< 11) male and female Nazca boobies.** Age: centered, continuous; FP: a dichotomous factor for Fish Phase; SSTA_AMJ_ and SSTA_DJF_: local sea surface temperature anomalies averaged across Apr-Jun and Dec-Feb, respectively; *El Niño*: a dichotomous factor marking the 1997-98 extreme ENSO warm event. SSTA_AMJ_ is not included as a predictor for traits expressed prior to April. The variance for the random effects associated with each model are presented along with the intraclass correlation coefficient (ICC; the proportion of total variance not accounted for by fixed factors) in brackets.

| **Male** | **Annual Breeding Success (19 yrs)** | | **Annual Breeding Success (12 yrs)** | | **p(lay \| alive) (12 yrs)** | | **p(hatch \| lay) (12 yrs)** | | **p(independent offspring \| hatch) (12 yrs)** | |
| --- | --- | --- | --- | --- | --- | --- | --- | --- | --- | --- |
| **Fixed effects** | **Estimate [95% CI]** | ***P*** | **Estimate [95% CI]** | ***P*** | **Estimate [95% CI]** | ***P*** | **Estimate [95% CI]** | ***P*** | **Estimate [95% CI]** | ***P*** |
| Intercept | -2.21 [-2.58, -1.81] | *** | -2.24 [-2.45, -1.98] | *** | -0.52 [-0.94, -0.09] | * | 0.66 [0.44, 0.89] | *** | 0.47 [0.21, 0.71] | *** |
| SSTA_AMJ_ | not in top model |  | -0.48 [-0.77, -0.20] | ** | na |  | na |  | -0.52 [-0.89, -0.15] | ** |
| SSTA_DJF_ | 0.43 [-0.01, 0.82] | . | 0.62 [0.38, 0.84] | *** | 0.78 [0.34, 1.30] | ** | 0.31 [0.03, 0.58] | * | 0.50 [0.17, 0.87] | ** |
| *El Niño* | -6.57 [-23.52, -4.40] | *** | -7.29 [-15.06, -5.81] | *** | -3.49 [-5.51, -1.78] | *** | -2.31 [-3.40, -1.22] | *** | -7.10 [-19.07, -5.33] | *** |
| FP (Sardine) | 1.33 [0.62, 2.02] | *** | 1.53 [1.20, 1.86] | *** | 1.19 [0.53, 1.80] | *** | 0.62 [0.28, 0.92] | *** | 1.34 [0.95, 1.74] | *** |
| Age | 0.54 [0.48, 0.57] | *** | 0.71 [0.62, 0.78] | *** | 0.67 [0.61, 0.72] | *** | 0.27 [0.19, 0.34] | *** | 0.28 [0.18, 0.38] | *** |
| Age^2^ | -0.06 [-0.08, -0.05] | *** | -0.07 [-0.10, -0.04] | *** | -0.04 [-0.06, -0.02] | *** | -0.03 [-0.07, 0.00] | * | not in top model |  |
| FP (Sardine):Age | not in top model |  | not in top model |  | not in top model |  | not in top model |  | not in top model |  |
| **Random effects** | **Variance [ICC]** |  | **Variance [ICC]** |  | **Variance [ICC]** |  | **Variance [ICC]** |  | **Variance [ICC]** |  |
| ID | 0.91 [0.16] |  | 1.09 [0.20] |  | 1.86 [0.29] |  | 0.23 [0.05] |  | 0.18 [0.04] |  |
| Year | 0.46 [0.08] |  | 0.03 [0.01] |  | 0.27 [0.04] |  | 0.04 [0.01] |  | 0.04 [0.01] |  |
| *R^2^_m_^1^* | 0.30 |  | 0.52 |  | 0.33 |  | 0.09 |  | 0.31 |  |
| *R^2^_c_^1^* | 0.50 |  | 0.64 |  | 0.60 |  | 0.16 |  | 0.35 |  |
| N cases | 15,884 |  | 5,038 |  | 5,038 |  | 2,343 |  | 1,563 |  |
|  |  |  |  |  |  |  |  |  |  |  |
| **Female** | **Annual Breeding Success (19 yrs)** | | **Annual Breeding Success (12 yrs)** | | **p(lay \| alive) (12 yrs)** | | **p(hatch \| lay) (12 yrs)** | | **p(independent offspring \| hatch) (12 yrs)** | |
| **Fixed effects** | **Estimate [95% CI]** | ***P*** | **Estimate [95% CI]** | ***P*** | **Estimate [95% CI]** | ***P*** | **Estimate [95% CI]** | ***P*** | **Estimate [95% CI]** | ***P*** |
| Intercept | -1.07 [-1.54, -0.58] | *** | -1.03 [-1.52, -0.47] | *** | 1.91 [1.39, 2.52] | *** | 0.78 [0.52, 1.05] | *** | 0.59 [0.23, 0.95] | ** |
| SSTA_AMJ_ | not in top model |  | not in top model |  | na |  | na |  | not in top model |  |
| SSTA_DJF_ | 0.57 [0.01, 1.08] | * | 1.11 [0.60, 1.72] | *** | 1.14 [0.39, 1.92] | ** | 0.48 [0.21, 0.78] | ** | 0.61 [0.18, 1.12] | ** |
| *El Niño* | -5.75 [-13.32, -2.87] | *** | -7.57 [-21.64, -5] | *** | -3.15 [-6.39, 0.20] | . | -2.43 [-3.54, -1.36] | *** | -6.71 [-9.46, -4.81] | *** |
| FP (Sardine) | 1.10 [0.16, 1.94] | * | 1.27 [0.46, 1.98] | ** |  |  | 0.66 [0.27, 1.02] | *** | 1.26 [0.71, 1.83] | *** |
| Age | 0.41 [0.38, 0.44] | *** | 0.41 [0.35, 0.48] | *** | 0.44 [0.37, 0.49] | *** | 0.21 [0.16, 0.26] | *** | 0.20 [0.12, 0.28] | *** |
| Age^2^ | -0.09 [-0.10, -0.07] | *** | -0.09 [-0.12, -0.07] | *** | -0.08 [-0.10, -0.05] | *** | -0.05 [-0.07, -0.03] | *** | -0.05 [-0.08, -0.02] | ** |
| FP (Sardine):Age | not in top model |  | not in top model |  | not in top model |  | not in top model |  | not in top model |  |
| **Random effects** | **Variance [ICC]** |  | **Variance [ICC]** |  | **Variance [ICC]** |  | **Variance [ICC]** |  | **Variance [ICC]** |  |
| ID | 0.46 [0.08] |  | 0.33 [0.07] |  | 0.76 [0.13] |  | 0.20 [0.04] |  | 0.08 [0.02] |  |
| Year | 0.66 [0.12] |  | 0.41 [0.08] |  | 0.77 [0.13] |  | 0.05 [0.01] |  | 0.14 [0.03] |  |
| *R^2^_m_^1^* | 0.24 |  | 0.42 |  | 0.29 |  | 0.12 |  | 0.29 |  |
| *R^2^_c_^1^* | 0.44 |  | 0.53 |  | 0.52 |  | 0.18 |  | 0.34 |  |
| N cases | 11,773 |  | 3,678 |  | 3,678 |  | 2,657 |  | 1,787 |  |

*P*: 0 ‘***’ 0.001 ‘**’ 0.01 ‘*’ 0.05 ‘.’ 0.1 ‘ ’ 1

*^1^*Marginal *R^2^* (*R^2^_m_*), and conditional *R^2^* (*R^2^_c_*) estimates were calculated following Nakagawa S, Schielzeth HA. A general and simple method for obtaining R^2^ from generalized linear mixed-effects models. Methods in Ecology and Evolution. 2013; 4: 133-142.
