# Supplementary material for: Decadal-scale variation in diet forecasts persistently poor breeding under ocean warming in a tropical seabird
Source: PLoS One. 2017 Aug 23;12(8):e0182545. doi: 10.1371/journal.pone.0182545 (PMC5568137; doi:10.1371/journal.pone.0182545)
Supplement: S8 Table — YBD: centered, continuous, years before death; FP: a dichotomous factor for Fish Phase; SSTAAMJ and SSTADJF: local sea surface temperature anomalies averaged across Apr-Jun and Dec-Feb, respectively. SSTAAMJ is not included as a predictor for traits expressed prior to April. The variance for the random effects associated with each model are presented along with the intraclass correlation coefficient (ICC; the proportion of total variance not accounted for by fixed factors) in brackets. (DOCX) [file pone.0182545.s014.docx]

**S8 Table. Coefficient estimates for the GLMM (binomial errors, logit link) best explaining variation (lowest AICc value; the “top model”) in Annual Breeding Success and sequential reproductive stages in presumed old male and female Nazca boobies.** YBD: centered, continuous, years before death; FP: a dichotomous factor for Fish Phase; SSTA_AMJ_ and SSTA_DJF_: local sea surface temperature anomalies averaged across Apr-Jun and Dec-Feb, respectively. SSTA_AMJ_ is not included as a predictor for traits expressed prior to April. The variance for the random effects associated with each model are presented along with the intraclass correlation coefficient (ICC; the proportion of total variance not accounted for by fixed factors) in brackets.

| **Male** | **Annual Breeding Success (18 yrs)** | | **Annual Breeding Success (11 yrs)** | | **p(lay \| alive) (11 yrs)** | | **p(hatch \| lay) (11 yrs)** | | **p(independent offspring \| hatch) (11 yrs)** | |
| --- | --- | --- | --- | --- | --- | --- | --- | --- | --- | --- |
| **Fixed effects** | **Estimate [95% CI]** | ***P*** | **Estimate [95% CI]** | ***P*** | **Estimate [95% CI]** | ***P*** | **Estimate [95% CI]** | ***P*** | **Estimate [95% CI]** | ***P*** |
| Intercept | -2.21 [-2.55, -1.74] | *** | -2.35 [-2.81, -1.86] | *** | -0.36 [-1.00, 0.26] |  | 0.59 [0.30, 0.85] | *** | -0.01 [-0.43, 0.40] |  |
| SSTA_AMJ_ | not in top model |  | not in top model |  | na |  | na |  | not in top model |  |
| SSTA_DJF_ | 0.37 [-0.10, 0.77] |  | 1.10 [0.56, 1.61] | *** | 0.85 [0.16, 1.46] | * | 0.63 [0.23, 1.02] | ** | 0.96 [0.40, 1.54] | *** |
| FP (Sardine) | 1.60 [0.75, 2.26] | *** | 1.92 [1.11, 2.49] | *** | 1.40 [0.37, 2.41] | ** | 0.92 [0.36, 1.42] | *** | 1.68 [1.02, 2.49] | *** |
| YBD | 0.20 [0.11, 0.28] | *** | 0.26 [0.12, 0.38] | *** | 0.38 [0.23, 0.51] | *** | not in top model |  | 0.21 [0.07, 0.37] | ** |
| FP(Sardine):YBD | not in top model |  | not in top model |  | not in top model |  | not in top model |  | not in top model |  |
| **Random effects** | **Variance [ICC]** |  | **Variance [ICC]** |  | **Variance [ICC]** |  | **Variance [ICC]** |  | **Variance [ICC]** |  |
| ID | 1.42 [0.23] |  | 1.46 [0.25] |  | 4.97 [0.51] |  | 0.33 [0.07] |  | 0.00 [0.00] |  |
| Year | 0.47 [0.08] |  | 0.21 [0.03] |  | 0.46 [0.05] |  | 0.05 [0.01] |  | 0.13 [0.03] |  |
| *R^2^_m_^1^* | 0.07 |  | 0.18 |  | 0.08 |  | 0.07 |  | 0.18 |  |
| *R^2^_c_^1^* | 0.41 |  | 0.46 |  | 0.65 |  | 0.16 |  | 0.21 |  |
| N cases | 3,175 |  | 1,568 |  | 1,568 |  | 753 |  | 515 |  |
|  |  |  |  |  |  |  |  |  |  |  |
| **Female** | **Annual Breeding Success (18 yrs)** | | **Annual Breeding Success (11 yrs)** | | **p(lay \| alive) (11 yrs)** | | **p(hatch \| lay) (11 yrs)** | | **p(independent offspring \| hatch) (11 yrs)** | |
| **Fixed effects** | **Estimate [95% CI]** | ***P*** | **Estimate [95% CI]** | ***P*** | **Estimate [95% CI]** | ***P*** | **Estimate [95% CI]** | ***P*** | **Estimate [95% CI]** | ***P*** |
| Intercept | -1.80 [-2.29, -1.19] | *** | -1.38 [-1.92, -0.80] | *** | 1.96 [1.29, 2.69] | *** | 0.42 [0.14, 0.70] | ** | 0.18 [-0.37, 0.72] |  |
| SSTA_AMJ_ | not in top model |  | not in top model |  | na |  | na |  | not in top model |  |
| SSTA_DJF_ | 0.60 [0.00, 1.23] | . | 1.26 [0.62, 1.96] | *** | 1.32 [0.66, 2.06] | *** | 0.61 [0.23, 0.99] | ** | 0.97 [0.27, 1.62] | ** |
| FP (Sardine) | 1.68 [0.72, 2.64] | ** | 1.45 [0.57, 2.35] | ** | 0.82 [-0.26, 1.98] |  | 0.87 [0.33, 1.41] | ** | 1.38 [0.42, 2.17] | ** |
| YBD | 0.21 [0.12, 0.30] | *** | 0.26 [0.14, 0.38] | *** | 0.43 [0.24, 0.63] | *** | 0.22 [0.12, 0.33] | *** | not in top model |  |
| FP(Sardine):YBD | not in top model |  | not in top model |  | -0.38 [-0.85, 0.02] | . | not in top model |  | not in top model |  |
| **Random effects** | **Variance [ICC]** |  | **Variance [ICC]** |  | **Variance [ICC]** |  | **Variance [ICC]** |  | **Variance [ICC]** |  |
| ID | 1.11 [0.18] |  | 0.81 [0.15] |  | 2.63 [0.36] |  | 0.26 [0.06] |  | 0.52 [0.10] |  |
| Year | 0.82 [0.13] |  | 0.43 [0.08] |  | 0.45 [0.06] |  | 0.07 [0.01] |  | 0.30 [0.06] |  |
| *R^2^_m_^1^* | 0.09 |  | 0.17 |  | 0.13 |  | 0.07 |  | 0.11 |  |
| *R^2^_c_^1^* | 0.43 |  | 0.40 |  | 0.55 |  | 0.15 |  | 0.28 |  |
| N cases | 2,548 |  | 1,144 |  | 1,144 |  | 908 |  | 582 |  |

*P*: 0 ‘***’ 0.001 ‘**’ 0.01 ‘*’ 0.05 ‘.’ 0.1 ‘ ’ 1

*^1^*Marginal *R^2^* (*R^2^_m_*), and conditional *R^2^* (*R^2^_c_*) estimates were calculated following Nakagawa S, Schielzeth HA. A general and simple method for obtaining R^2^ from generalized linear mixed-effects models. Methods in Ecology and Evolution. 2013; 4: 133-142.
